# An assessment of executive function in two different rat models of attention-deficit hyperactivity disorder: Spontaneously hypertensive versus *Lphn3* knockout rats

**DOI:** 10.1111/gbb.12767

**Published:** 2021-09-08

**Authors:** Helen J. K. Sable, Deranda B. Lester, Joshua L. Potter, Hunter G. Nolen, Destinee M. Cruthird, Lauren M. Estes, Alyssa D. Johnson, Samantha L. Regan, Michael T. Williams, Charles V. Vorhees

**Affiliations:** 1 Department of Psychology, University of Memphis, Memphis, Tennessee, USA; 2 Department of Pediatrics, University of Cincinnati College of Medicine, Cincinnati, Ohio, USA; 3 Division of Neurology, Cincinnati Children’s Hospital Medical Center, Cincinnati, Ohio, USA

**Keywords:** *Adgrl3*, ADHD, CRISPR/Cas9, delayed spatial alternation, differential reinforcement of low rates, externalizing behavior, inhibitory control, Latrophilin-3, *Lphn3*, working memory

## Abstract

Attention-deficit/hyperactivity disorder (ADHD) a common neurodevelopmental disorder of childhood and often comorbid with other externalizing disorders (EDs). There is evidence that externalizing behaviors share a common genetic etiology. Recently, a genome-wide, multigenerational sample linked variants in the *Lphn3* gene to ADHD and other externalizing behaviors. Likewise, limited research in animal models has provided converging evidence that *Lphn3* plays a role in EDs. This study examined the impact of *Lphn3* deletion (i.e., *Lphn3*^−*/*−^) in rats on measures of behavioral control associated with externalizing behavior. Impulsivity was assessed for 30 days via a differential reinforcement of low rates (DRL) task and working memory evaluated for 25 days using a delayed spatial alternation (DSA) task. Data from both tasks were averaged into 5-day testing blocks. We analyzed overall performance, as well as response patterns in just the first and last blocks to assess acquisition and steady-state performance, respectively. “Positive control” measures on the same tasks were measured in an accepted animal model of ADHD–the spontaneously hypertensive rat (SHR). Compared with wildtype controls, *Lphn3*^−*/*−^ rats exhibited deficits on both the DRL and DSA tasks, indicative of deficits in impulsive action and working memory, respectively. These deficits were less severe than those in the SHRs, who were profoundly impaired on both tasks compared with their control strain, Wistar-Kyoto rats. The results provide evidence supporting a role for *Lphn3* in modulating inhibitory control and working memory, and suggest additional research evaluating the role of *Lphn3* in the manifestation of EDs more broadly is warranted.

## 1 | INTRODUCTION

Attention deficit/hyperactivity disorder (ADHD) is characterized by persistent problems with inattention, impulsivity, and/or hyperactivity lasting for at least 6 months and resulting in functional impairment in a variety of settings.^1^ Based on the 2016 National Survey of Children’s Health an estimated 9.4% of all US children (2–17 years of age) received an ADHD diagnosis^2^ making it the most common neurodevelopmental disorder diagnosed in childhood.^3,4^ ADHD can persist into adulthood and is often comorbid with other externalizing disorders (EDs) such as oppositional defiant disorder and conduct disorder, which typically present during childhood or early adolescence,^1,5^ as well as antisocial personality disorder and substance use disorder (SUD) which usually manifest during late adolescence or young adulthood.^6,7^

In addition, to documented shared liability for EDs,^8–11^ there is also evidence that externalizing behaviors may stem from a common genetic etiology.^12,13^ While the heritability of ADHD alone is high (*h*^2^ = 74%),^14^ much of it is common to other EDs, so much so that a single highly heritable (*h*^*2*^ ~ 80%) externalizing factor accounts for a larger percentage of the genetic variation than ADHD alone.^12,15^ Data from a genome-wide, multigenerational sample identified linkage of ADHD as well as co-segregation of ADHD and other externalizing behaviors with markers on chromosome 4q13.2.^16–19^ Mapping of this region revealed variants in the *Lphn3* (*Adgrl3*) gene [OMIM 616417] that predisposed individuals to ADHD,^20–22^ predicted ADHD severity and response to treatment,^22–24^ and predicted SUD in ADHD patients.^25^ Similar studies in other populations replicated the finding that *Lphn3* gene variants contribute to ADHD susceptibility.^26–32^ In addition, in SUD patients, *Lphn3* was one of 86 “risk genes” identified.^33^

Research in animal models has provided converging evidence that the *Lphn3* gene plays a role in EDs.^34^ For example, *Lphn3*^−/−^ knockout (KO) mice exhibit hyperactivity in the open field test, increased premature responses (indicative of impulsivity) on a continuous performance test, and are more sensitive to the locomotor stimulant effects of cocaine.^35,36^ Similarly, *Lphn3* KO rats were also hyperactive,^37^ while zebrafish that lacked *lphn3.1* (one of two *lphn3* zebrafish orthologs) exhibited increased locomotion, nighttime hyperactivity, and episodes of motor impulsivity.^38^

Research evaluating the impact of *Lphn3* gene expression variants on behavioral assays associated with EDs is limited. The present experiments examined the impact of *Lphn3* deletion on operant measures of behavioral control including impulsivity and working memory. Impulsivity is a core symptom of ADHD^1^ and is also a known risk factor for SUD.^39–41^ Increasing working memory load has been argued to increase impulsive behavior.^42,43^ Not surprisingly, working memory deficits are commonly reported in ADHD children^44–47^ and are associated with drug-seeking in rats^48–51^ and SUD in humans.^52–54^ In the current study, impulsivity was assessed for 30 days, while working memory was assessed for 25 days. To determine the suitability of *Lphn3*^−*/*−^ (KO) rats as a prospective animal model of ADHD and assess their relative degree of impairment, we also report “positive control” measures of the same behaviors in Spontaneously Hypertensive Rats (SHRs) as numerous studies have shown their utility as an animal model of ADHD.^55–59^ In line with previous research, we expected to find an overall deficit in both impulsive action and working memory in the SHRs relative to Wistar-Kyoto (WKY) rats (i.e., the SHR control strain) as well as in the *Lphn3* KO rats relative to their *Lphn3*^+*/*+^ (WT) controls. Additionally, we examined whether observed deficits in performance occurred during response acquisition and/or during steady-state performance.

## 2 | MATERIAL AND METHODS

### 2.1 | Subjects

The subjects were 24 SHRs (12 male, 12 female) and 24 WKY (12 male, 12 female) rats purchased from Charles River (Kingston, NY) as well as 17 *Lphn3*^−*/*−^ (eight male, nine female) and 18 *Lphn3*^+*/*+^ (nine male, nine female) rats on a Sprague Dawley background (SD-IGS, strain 001, Charles River, Charleston, NC). The SHR and WKY rats were shipped to the University of Memphis when they were 45 ± 2 days old. The *Lphn3*^−*/*−^ rats were generated at the Cincinnati Children’s Transgenic Animal and Genome Editing Core by using CRISPR/Cas9 to delete exon 3.^37^ The *Lphn3*^−*/*−^ founders were then bred with *Lphn3*^+*/*+^ rats to establish the lines and heterozygote crossings (i.e., *Lphn3*^+*/*−^ x *Lphn3*^+*/*−^) were used to generate the KO and WT rats used for these experiments. Ear punches were collected from offspring at postnatal day (P)7 for genotyping using three primers: (1) AAAGGGTCATAGCATCCGGC, (2) CTAACGTGGCTTTTTGTCTTCT, and (3) GCTCGACAGACAGTGTGGAT. HotStarTaq Master Mix kit (Qiagen, Hilden, Germany) was used per manufacturer’s recommendations. Thermocycler parameters were: (1) 95°C, 5 min; (2) 94°C, 1 min; (3) 61.5°C, 1 min; (4) 72°C, 2 min; (5) steps 2–4 were repeated 34 more times, followed by (6) 72°C, 10 min; and (7) held at 4°C until the product was run on a 2% agarose gel with ethidium bromide staining. The WT band appears at ~320 bp and the KO band at ~452 bp.^37^ Only one rat per genotype per sex per litter was randomly selected and shipped to the University of Memphis when 30 ± 2 days old.

Upon arrival at the University of Memphis Psychology vivarium, same-sex rats of the same strain or the same genotype were housed 2–3 per cage in standard plastic cages (45 cm × 24 cm × 30 cm) with corn cob bedding and ad libitum tap water in a room with a 12 h reverse light/dark cycle (lights off 0700 h) that was temperature- and humidity-controlled. Rats remained on free feed (Teklad, 2018) until all rats in a shipment reached P60 after which they were put on a food restriction schedule to maintain 85%–90% of their free-feeding weight. Adjustments were made bi-weekly to account for growth. All animal use was approved by the Institutional Animal Care and Use Committee at the University of Memphis and was in accordance with Public Health Service Policy on Humane Care and Use of Laboratory Animals.^60^ Personnel were blinded to the genotype or strain of the rats during behavioral testing.

### 2.2 | Apparatus

Behavioral tests were performed in automated, rat operant chambers (Med Associates Inc., St. Albans, VT) housed in sound attenuated wooden boxes. The test chambers were 17.5 cm tall with a 24 cm × 20 cm stainless steel grid floor that rests above a tray filled with corn cob bedding. Dustless precision pellets (BioServ product F0165) were dispensed into a food magazine centered 2.5 cm above the floor. A retractable lever with a cue light located above was located on each side of the food magazine and a house light was located on the wall opposite the food magazine. White noise was presented during testing to mitigate the intrusion of outside sounds. Med-PC V software was used to present the testing programs and record data.

### 2.3 | Procedure

When all rats in a shipment reached P70, behavioral testing began and followed the testing sequence below. Rats received one session/day and were tested at the same time of day 0800–1100 h, 7 days/week.

#### 2.3.1 | Autoshaping

This program is designed for rats to learn the basics of the operant chamber, such as the location of food and the association between lever presses and food delivery. Upon activation of the autoshaping program, two levers were extended. When a lever was pressed, a pellet was dispensed into the magazine. If neither lever was pressed within a span of 3 min, a free pellet was dispensed. The program ended after 60 min or after 100 pellets were dispensed. Rats remained on autoshaping until no free pellets were delivered (~2–3 sessions).

#### 2.3.2 | Fixed ratio training

This program strengthened the lever-press response and ensured there was not a side preference for either lever. At the start of each fixed ratio (FR) session, the right lever was extended and the right cue light was illuminated. As with autoshaping, each lever press on an extended lever resulted in a food pellet. However, after five pellets were dispensed, the right lever was retracted and the right cue light turned off. At the same time, the left lever was extended and left cue light activated until five reinforcers were delivered. The response requirement then returned to the other side and this pattern of five lever presses followed by alternation was repeated until 100 total reinforcers were delivered. FR training lasted for three sessions.

#### 2.3.3 | Differential reinforcement of high rates

For differential reinforcement of high rates (DRH), only the left response lever was extended. To earn a reinforcer, the rat was required to make a minimum number of responses within a specific time interval. For example, during DRH 2:1 a reinforcer was delivered if two responses were made within 1 s. Only three sessions of DRH testing were conducted, including 1 day of DRH 2:1, 1 day of DRH 4:2, and one day of DRH 8:4. Each session terminated after 200 reinforcers were delivered or 90 min had elapsed, whichever occurred first.

#### 2.3.4 | Differential reinforcement of low rates

Three differential reinforcement of low rates (DRL) programs were conducted. At the start of each trial, the right lever was extended and the rat was required to press the lever to start the response timer. Once pressed, the rat had to wait for a set inter-response time (IRT) to elapse before pressing the lever again. If the lever was pressed after the set IRT elapsed, a food pellet was dispensed. However, if the lever was pressed before the allotted IRT passed, no reinforcer was delivered, and the rat was required to press the lever again to start a new trial. The IRTs included 2 days with a 5 s IRT (DRL 5), 2 days with a 10 s IRT (DRL 10), and 30 days with a 15 s IRT (DRL 15).

#### 2.3.5 | Cued alternation

Rats were trained to alternate their lever presses from one lever to the other from trial to trial. At the beginning of each cued alternation (CA) session, both levers were extended and both cue lights illuminated. Once a lever was pressed, the levers retracted and the alternate cue light was illuminated and both levers re-extended. To receive a reinforcer, the rat had to press the lever below the illuminated cue light, which alternated from one trial to the next. There was no delay imposed between trails except for the time needed for the levers to retract and re-extend at the start of the next trial. The rats completed 200 trials in each session. Rats remained on CA, until they met a performance criterion of 60% correct.

#### 2.3.6 | Noncued alternation

Rats had to alternate from one lever to the next from one trial to the next but did so without the assistance of cue lights. Each noncued alternation (NCA) session had 200 trials. Ten NCA sessions were conducted.

#### 2.3.7 | Delayed spatial alternation

After NCA, rats were tested on delayed spatial alternation (DSA) for 25 days. This task was identical to NCA except that a delay of 0, 5, 10, or 20 s was imposed between trials. These delays were presented randomly with the exception that a given delay never occurred consecutively for more than three trials. All delays were equally distributed throughout a session such that each delay was presented 50 times for a total of 200 trials.

### 2.4 | Design and analyses

Data were analyzed using SPSS (IBM) version 26.0.

#### 2.4.1 | Differential reinforcement of high rates

Three different dependent measures were analyzed for DRH including total number of lever presses, reinforcers earned, and efficiency (reinforcers earned × DRH response requirement ÷ total number of lever presses). Each dependent measure was analyzed using a 2 (genotype or strain) × 2 (sex) × 3 (schedule) mixed ANOVA where genotype and sex were between-subjects factors and schedule (i.e., 2:1, 4:2, and 8:4) was a repeated measures factor.

#### 2.4.2 | Differential reinforcement of low rates

For DRL 5 and DRL 10, the ratio of reinforced to nonreinforced lever presses as well as the total number of reinforcers dispensed and number of trials completed were analyzed separately using a 2 (genotype or strain) × 2 (sex) × 2 (day) mixed ANOVA with day a repeated-measures factor. For the 30 days of DRL 15, the analyses were similar except that each dependent measure was averaged into 6, 5-day testing blocks and included in the mixed ANOVA as the repeated measure factor. Weincluded all blocks in the analyses of the ratio of reinforced to nonreinforced lever presses, total number of reinforcers dispensed, and the number of trials completed during DRL 15. This was done to assess whether genotype or strain affected task acquisition (i.e., how quickly the task was learned) and/or steady-state responding (i.e., how well the task was learned).

#### 2.4.3 | DRL response pattern analysis

Response pattern analysis was conducted whereby the proportions of responses that fell within 2.5 s IRT bins were analyzed via a 2 (genotype or strain) × 2 (sex) × 2 (day or block) × (IRT bin) mixed ANOVA. IRT bins were 2.5 s in size. For DRL 5, there were four: <2.5 s, 2.5–5.0 s, 5.0–7.5 s, and > 7.5 s. In a similar fashion, DRL 10 had 6 IRT bins and DRL 15 had 8 IRT bins. DRL 5 and 10 included both days in the analysis. To simplify interpretation but allow for response pattern analysis during acquisition and steady-state responding, DRL 15 included the first testing block (i.e., days 1–5) and the last testing block (i.e., days 25–30). Note that only the last two IRT bins for each DRL schedule were reinforced. Lastly, peak deviation analyses^61–63^ including the parameters of burst ratio, peak location, and peak area were also conducted on the last block of DRL 15 data. Each parameter was analyzed separately using a 2 (genotype or strain) × 2 (sex) between-subjects ANOVA.

#### 2.4.4 | Cued alternation

For CA, the number of sessions to reach criterion (i.e., 60% correct) was analyzed with a 2 (genotype or strain) × 2 (sex) between-subjects ANOVA.

#### 2.4.5 | Noncued alternation

The percent correct responses during NCA was analyzed using a 2 (genotype or strain) × 2 (sex) × 10 (day) mixed ANOVA which included day as a repeated-measures factor.

#### 2.4.6 | Delayed spatial alternation

The percent correct responses during DSA was analyzed similarly to NCA except that data over the 25 days of testing were averaged into 6, 5-day testing blocks and block was included in the mixed ANOVA as the repeated measure factor instead of day. Again, we included all blocks in the analysis of the percent correct during DSA to assess task acquisition and steady-state performance.

#### 2.4.7 | DSA trial-by-trial analyses

The types of errors made during DSA task acquisition (block 1) and steady-state performance (block 5) were also analyzed. These included win-stay errors and lose-stay errors. A “win” was defined as a correct response and happened when a rat correctly alternated levers. If the rat then “stayed” on the same lever, as the previous trial, this generated an incorrect response. Thus, a win–stay error indicated that the rat responded correctly on the n-1 trial but incorrectly on the nth trial by failing to alternate between the two levers.^64^ In addition, a “lose” was defined as a trial in which the rat responded incorrectly because it failed to alternate. Thus, a lose–stay error indicated that the rat responded incorrectly on the n-1 trial, and also on the nth trial by staying on the same lever. Therefore, a lose–stay error represents at least three consecutive responses on the same lever and is considered a perseverative response.^64^ Win-stay and lose-stay errors were analyzed separately using a 2 (genotype or strain) × 2 (sex) × 2 (first block, fifth block)mixed ANOVA.

## 3 | RESULTS

Two female SHRs were euthanized for health reasons prior to completion of NCA and DSA. If a sphericity violation was found for any within-subjects effect, a Greenhouse–Geisser correction was used to reduce the risk of a Type I error because ε < 0.75 in all cases.^65^ In the interest of brevity, detailed explanation of the significant genotype-, strain-, or sex-related main effects and interactions are only reported for DRL 15 and DSA as these were our primary assessments of impulsive action and working memory. However, supplemental figures are included detailing the statistical findings for DRH, DRL 5, DRL 10, CA, and NCA. Briefly, for DRH, significant genotype and strain differences were observed wherein female KO and SHRs of both sexes performed better than controls when a high response rate exclusive of response inhibition was required (Supplemental Figure 1). However, KO rats and SHRs exhibited impaired performance during DRL 5 (Supplemental Figures 2–3) and DRL 10 (Supplemental Figures 4–6), which both required response inhibition. Supplemental Figure 7 shows the “debursted” relative proportion of responses for the pause IRTs. No genotype or strain differences were observed during cued alternation (Supplemental Figure 8) when the correct response lever was illuminated. Surprisingly, while the KO and WT rats did not differ during NCA (i.e., correct lever not illuminated), SHRs exhibited impaired performance compared with WKY rats across all 10 days of testing (Supplemental Figure 9) even though there was no delay between lever retractions at the end of a trial to re-extension for the next trial.

### 3.1 | DRL 15

#### 3.1.1 | Ratio of reinforced to nonreinforced responses

**FIGURE 1:**
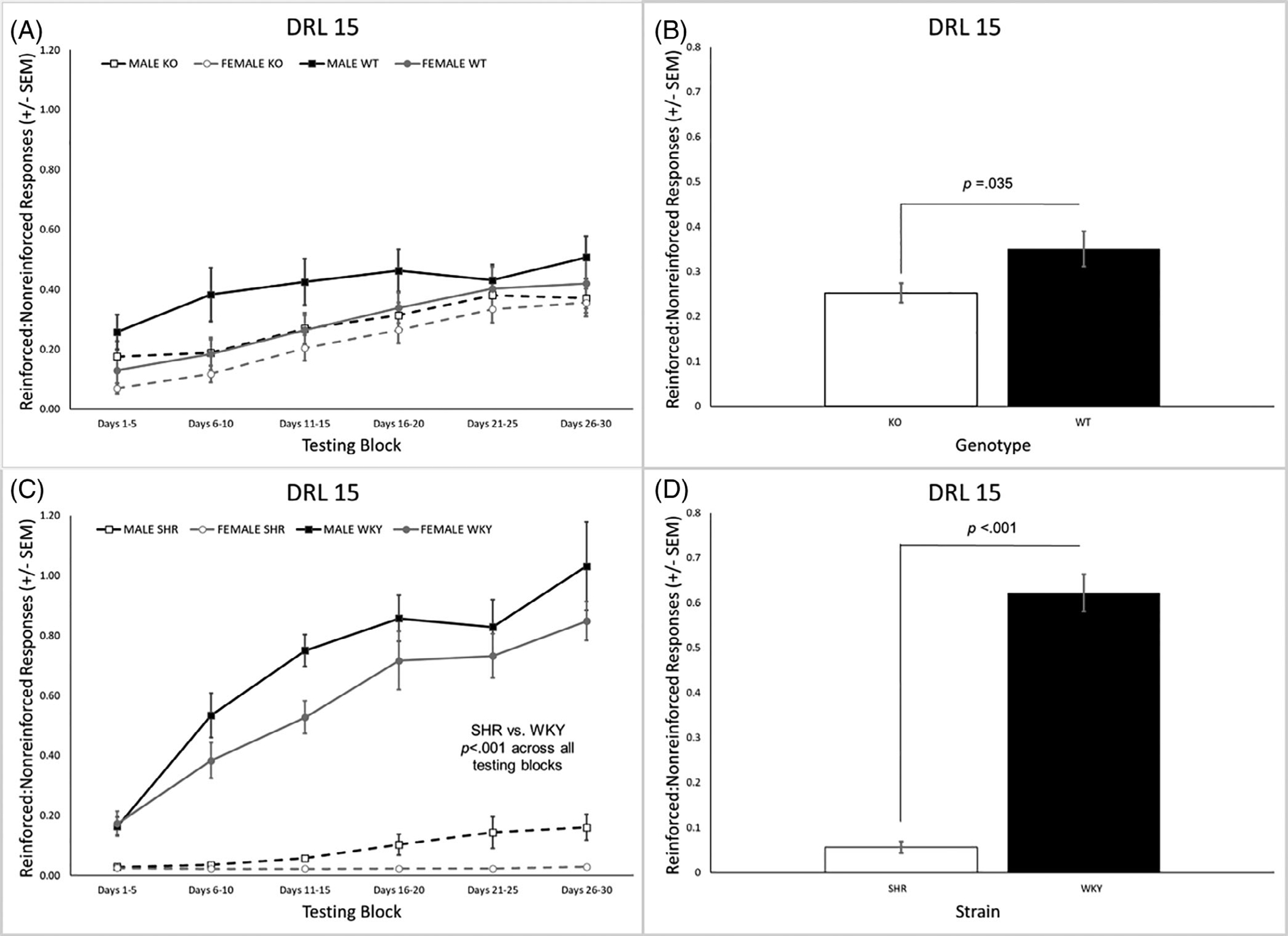
(A) The ratio of reinforced to nonreinforced trials across all testing blocks of DRL 15 for the *Lphn3* KO and WT rats. (B) *Lphn3* KO rats had a significantly lower ratio of reinforced to nonreinforced trials than the WT rats. (C) The ratio of reinforced to nonreinforced trials was significantly lower for the SHRs than the WKY within each of the six testing blocks. (D) SHRs had a significantly lower ratio of reinforced to nonreinforced trials than WKY rats overall. DRL, differential reinforcement of low rates; KO, knockout; SEM, standard error of the mean; SHR, spontaneously hypertensive rat; WKY, Wistar-Kyoto; WT, wildtype

The ratio of reinforced to nonreinforced trials across all DRL 15 testing blocks is presented in Figure 1A for the KO and WT rats. Higher values indicate better performance. There was a significant main effect of genotype, [*F*(1, 31) = 4.84, *p* = 0.035] and of sex [*F*(1, 31) = 4.18, *p* = 0.049]. KO rats had a lower ratio of reinforced: nonreinforced trials than WT rats (Figure 1B) and males had a higher ratio than females. The reinforced: nonreinforced ratio across all blocks for the SHR/WKY rats is presented in Figure 1C. There was a significant main effect of strain [*F*(1, 44) = 190.4, *p* < 0.001] and of sex [*F*(1, 44) = 5.5, *p* = 0.024]. Males had a higher ratio than females and the SHRs had a lower overall ratio than the WKY rats (Figure 1D). The strain × block interaction was also significant [*F*(1.8, 134.8) = 34.58, *p* < 0.001]. The SHRs had a significantly lower ratio than the WKY rats in every block (Figure 1C) with the magnitude of the difference increasing across blocks (Figure 1C).

#### 3.1.2 | Reinforcers earned

**FIGURE 2:**
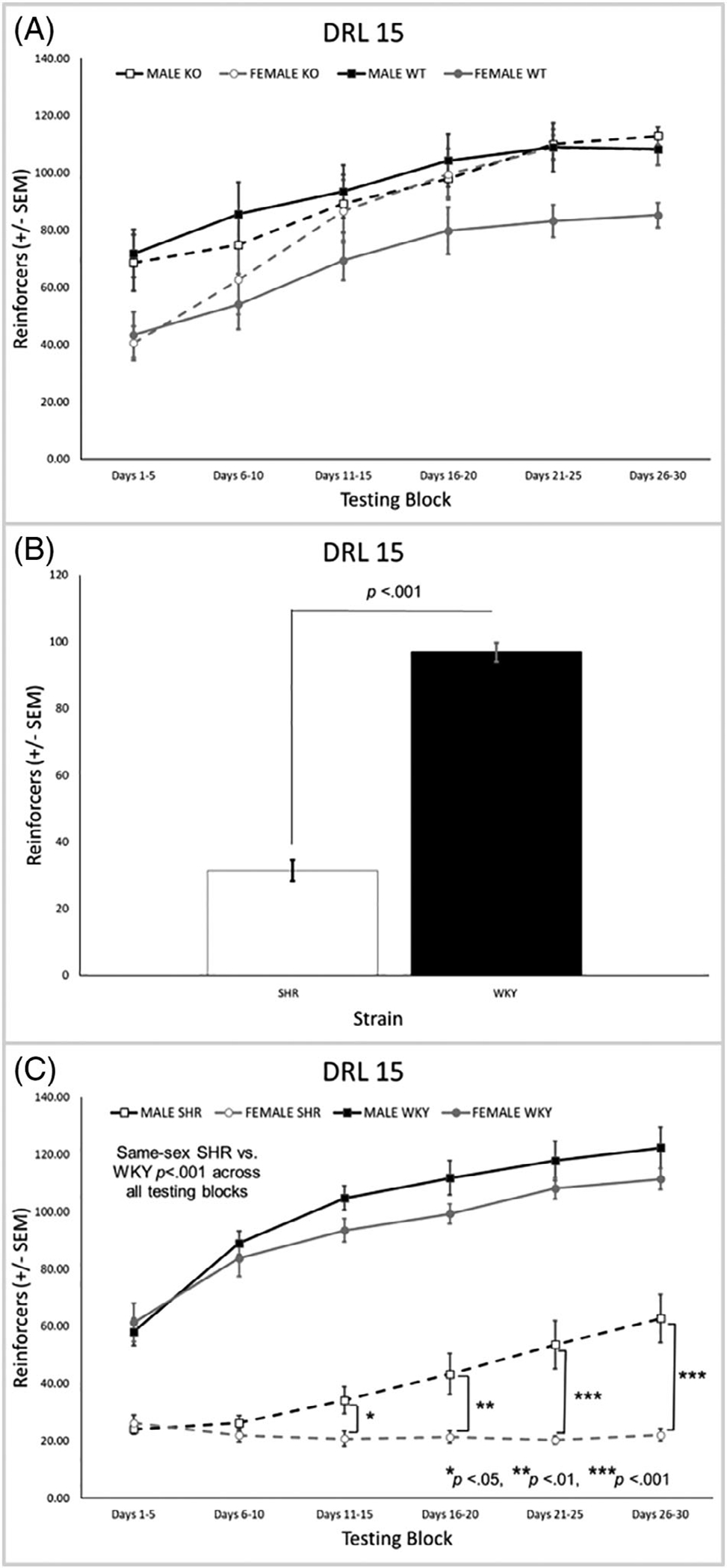
(A) There were no differences between the *Lphn3* KO and WT rats on reinforcers earned, but there was a significant main effect of sex with males earning more than females (*p* = 0.015). (B) SHRs earned fewer reinforcers overall than WKY rats. (C) SHR males earned significantly fewer reinforcers than WKY males with a similar effect in the females. In addition, SHR females earned fewer reinforcers than SHR males in blocks 3–6. DRL, differential reinforcement of low rates; KO, knockout; SEM, standard error of the mean; SHR, spontaneously hypertensive rat; WKY, Wistar-Kyoto; WT, wildtype

The main effect of genotype on reinforcers earned was not significant, but the main effect of sex was [*F*(1, 31) = 6.65, *p* = 0.015], with males earning more than females (Figure 2A). For the SHR/WKY rats, there were significant main effects of strain [*F*(1, 44) = 303.94, *p* < 0.001] and sex [*F*(1, 44) = 12.28, *p* = 0.001] and significant strain × block [*F*(2.2, 96.2) = 20.16, *p* < 0.001] and strain × sex × block [*F*(2.2, 96.2) = 3.34, *p* = 0.035] interactions. As can be seen in Figure 2B, SHRs earned fewer reinforcers overall than the WKY rats. This was driven by the finding that SHR males earned significantly fewer reinforcers than WKY males (*p* < 0.001) in all testing blocks, with a similar effect in the females (*p* < 0.001; Figure 2C). In addition, SHR females earned fewer reinforcers than SHR males during blocks 3–6 (Figure 2C).

#### 3.1.3 | Total number of trials completed

**FIGURE 3:**
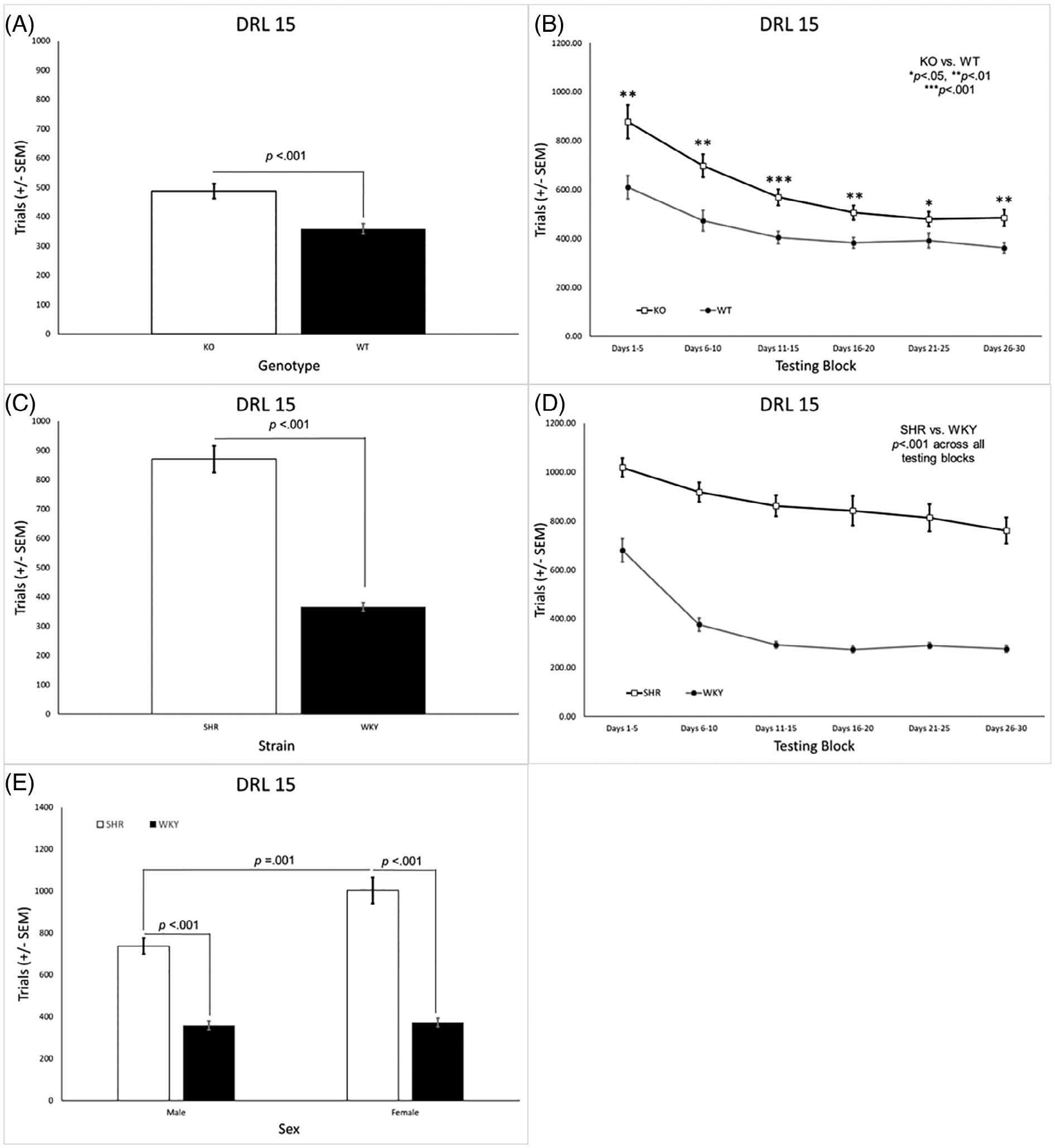
(A) *Lphn3* KO rats completed a significantly higher number of trials than the WT rats overall. (B) The difference between *Lphn3* KO and WT rats was present in all testing blocks. (C) SHRs completed significantly more trials then WKY rats. (D) The difference between SHR and WKY rats was present in all testing blocks. (E) SHR males and females completed more trials than their same-sex WKY counterparts. SHR females also completed more trials than SHR males. DRL, differential reinforcement of low rates; KO, knockout; SEM, standard error of the mean; SHR, spontaneously hypertensive rat; WKY, Wistar-Kyoto; WT, wildtype

There was a significant main effect of genotype [*F*(1, 31) = 19.22, *p* < 0.001] and a significant genotype × block interaction [*F*(2.4, 73.8) = 3.15, *p* = 0.040]. KO rats completed a higher number of trials than the WT rats overall (Figure 3A) and in all testing blocks (Figure 3B). There was also a significant main effect of sex [*F*(1, 31) = 4.83, *p* = 0.036] where females completed a significantly higher number of trials than males [data not shown]. For the SHR/WKY rats, there were significant main effects of strain [*F*(1, 44) = 162.8, *p* < 0.001] and sex [*F*(1, 44) = 12.61, *p* = 0.001] as well as significant strain × sex [*F*(1, 44) = 10.16, *p* = 0.003] and strain × block [*F*(2.3, 101.4) = 8.24, *p* < 0.001] interactions. SHRs completed significantly more trials than WKY rats overall (Figure 3C)–an effect that was present in each testing block (Figure 3D) and evident for each sex (Figure 3E). Females completed significantly more trials than males overall, particularly SHR females versus SHR males (Figure 3E).

#### 3.1.4 | Response pattern analysis

**FIGURE 4:**
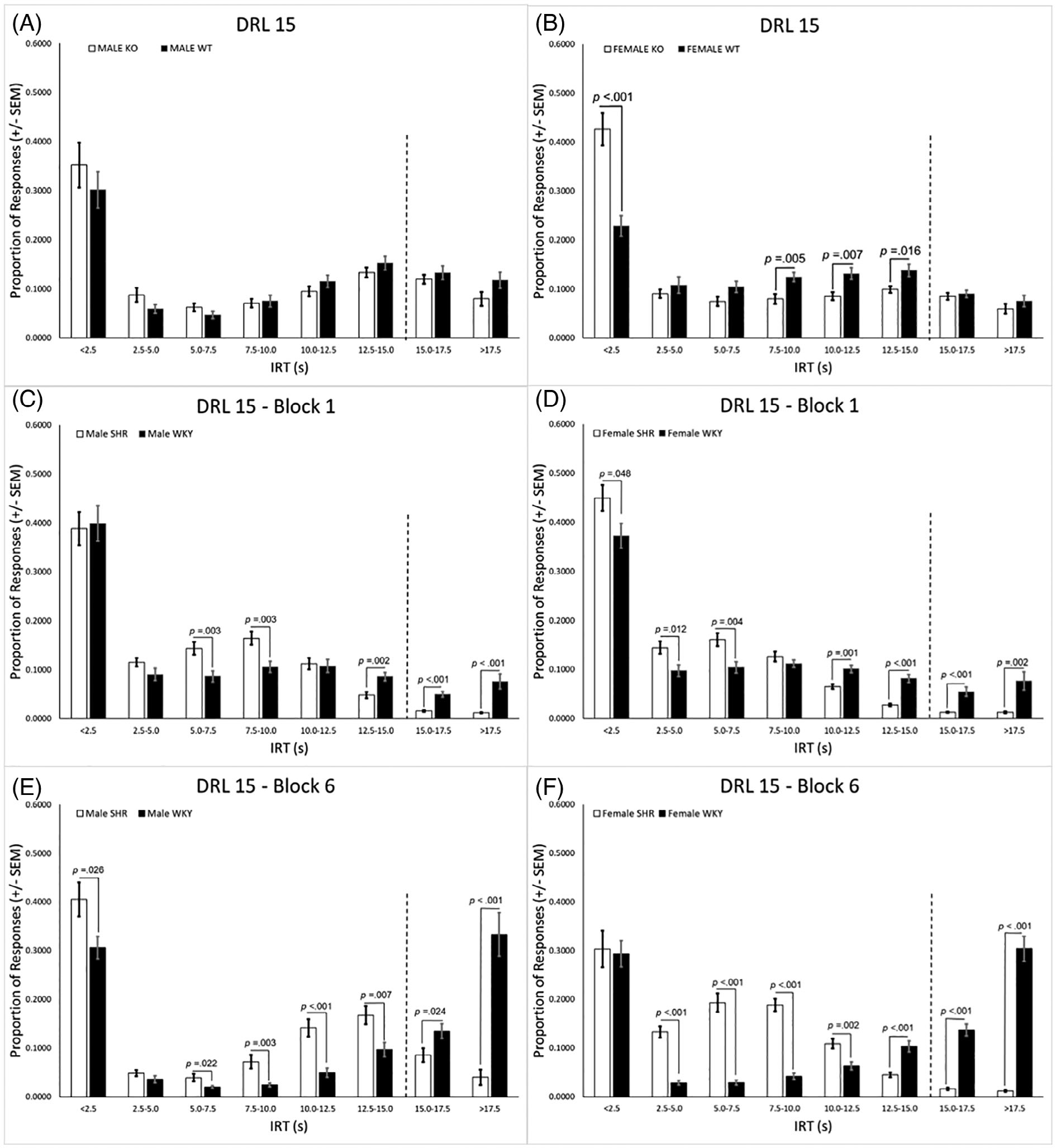
(A) The genotype x IRT interaction was not significant in the males. (B) Compared with WT females, KO females exhibited a significantly higher proportion of burst responses in the shortest IRT bin, but a lower proportion of responses in bins ranging from 7.5–15.0 s. (C) In block 1, SHR males had a higher proportion of responses in intermediate IRT bins ranging from 5.0–10.0 s, but a lower proportion of responses in IRT bins greater than 12.0 s when compared with WKY males. (D) SHR females in block 1 had a significantly higher proportion of responses than WKY females in the IRT bins that were less than 7.5, but a lower proportion of responses in IRT bins greater than 10.0 s. (E) In block 6 the SHR males exhibited a significantly higher proportion of responses than WKY males in all but one of the IRT bins less than 15.0 s, as well as a lower proportion of responses in both bins greater than 15.0 s. (F) SHR females in block 6 exhibited a significantly higher proportion of responses than WKY females in all bins ranging from 2.5–12.5 s, but a significantly lower proportion in the three longest IRT bins. DRL, differential reinforcement of low rates; KO, knockout; SEM, standard error of the mean; SHR, spontaneously hypertensive rat; WKY, Wistar-Kyoto; WT, wildtype

The genotype × IRT [*F*(2.2, 67.6) = 9.59, *p* < 0.001] and genotype × sex × IRT [*F*(2.2, 67.6) = 3.68, *p* = 0.027] interactions were significant. Post hoc analyses done separately for each sex revealed a significant genotype × IRT interaction in the females (*p* < 0.001) but not males (Figure 4A,B, respectively). Compared with WT females, KO females exhibited a significantly higher proportion of burst responses in the shortest IRT bin (i.e., <2.5 s), but a lower proportion of responses in IRT bins ranging from 7.5–15.0 s.

Response pattern analysis in the SHR/WKY rats revealed significant interactions of strain × block [*F*(1, 44) = 12.56, *p* = 0.001], strain × IRT [*F*(3.6, 160.4) = 40.17, *p* < 0.001], strain × sex × IRT [*F*(6.6, 160.4) = 6.59, *p* < 0.001], strain × block × IRT [*F*(3.8, 165.0) = 26.37, *p* < 0.001], and strain × sex × block × IRT [*F*(3.8165.0) = 3.28, *p* = 0.015]. Separate post hoc analyses were conducted for each sex and testing block. A significant genotype × IRT interaction was found in both the males (*p* = 0.046) and females (*p* < 0.001) in block 1. Compared with WKY males, SHR males had a higher proportion of responses in intermediate IRT bins ranging from 5.0–10.0 s, but a lower proportion of responses in longer IRT bins ≤12.0 s (Figure 4C). SHR females in block 1 had a significantly higher proportion of responses than WKY females in all IRT bins <7.5 s, but a lower proportion of responses in IRT bins ≤10.0 s (Figure 4D). A significant genotype × IRT interaction was also found for the males (*p* < 0.001) and females (*p* < 0.001) in block 6. The SHR males had a significantly higher proportion of responses than WKY males in all but one of the IRT bins <15.0 s, as well as a lower proportion of responses in bins >15.0 s (Figure 4E). A similar trend in block 6 was observed in the SHR females who had a significantly higher proportion of responses than WKY females in all bins ranging from 2.5–12.5 s, but a significantly lower proportion in the three longest IRT bins ≥12.5 s; Figure 4F).

#### 3.1.5 | Peak deviation analysis

**FIGURE 5:**
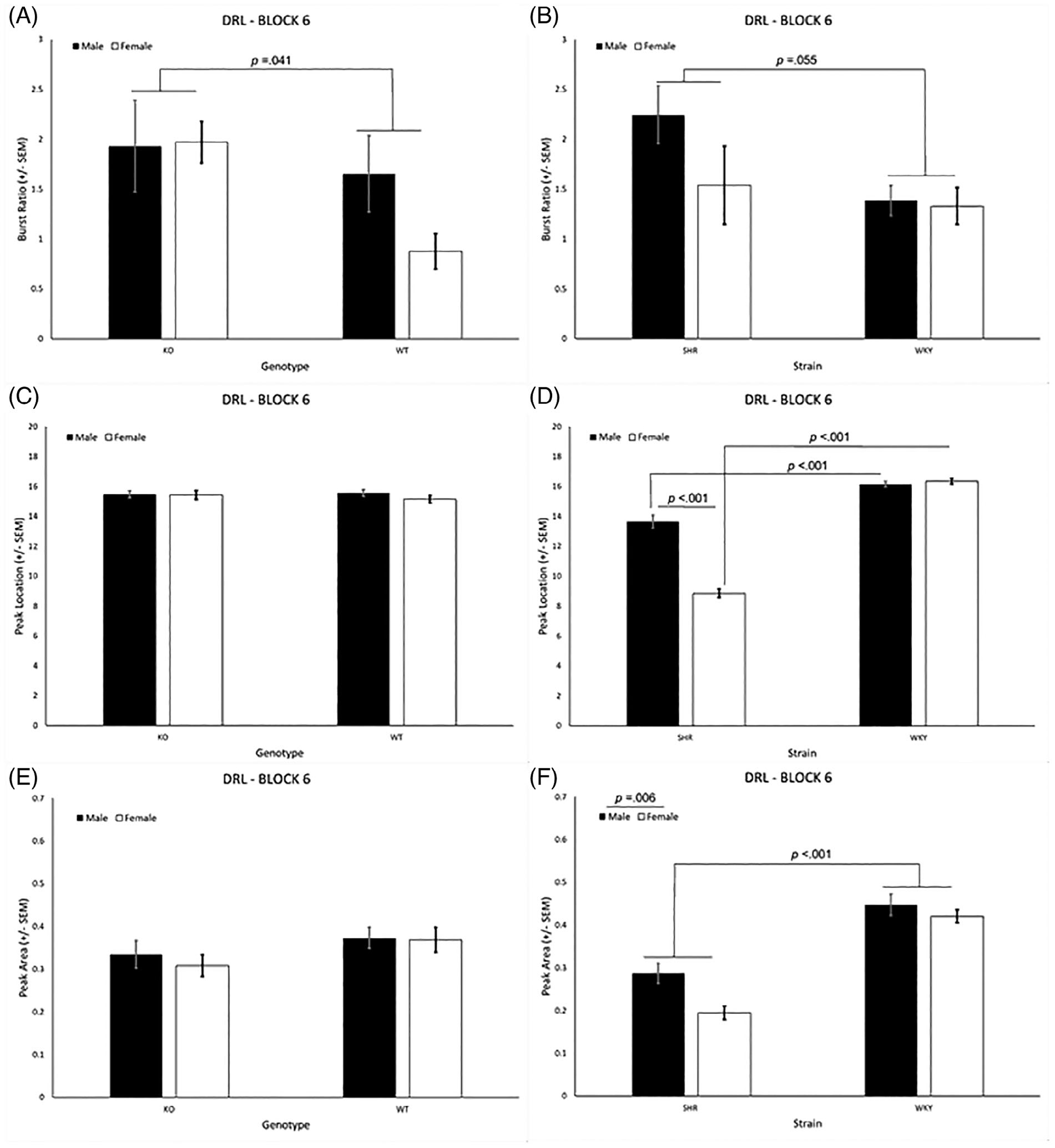
(A) The *Lphn3* KO rats exhibited a significantly higher burst ratio than the WT rats. (B) The SHRs exhibited a significantly higher burst ratio than the WKY rats. (C) Genotype did not affect peak location. (D) Peak location occurred sooner in the SHR males and females compared with their same-sex WKY counterparts. In addition, the peak location of SHR females occurred earlier than for SHR males. (E) Genotype did not affect peak area. (E) The peak area was larger in the WKY rats than the SHRs. DRL, differential reinforcement of low rates; KO, knockout; SEM, standard error of the mean; SHR, spontaneously hypertensive rat; WKY, Wistar-Kyoto; WT, wildtype

The negative exponential curves and the “debursted” relative proportion of responses for the pause IRTs (i.e., IRTs >2.5 s) for each group, as well as graphs showing the difference curves for the different genotypes and different strains used for peak deviation analysis [see 62, 63], are included in Supplemental Figure 7. Only responses that were < 2.5 s were considered burst responses, while all others were considered pause responses. The analyses for burst ratio revealed a significant main effect of genotype [*F*(1, 31) = 4.55, *p* = 0.041]. The *Lphn3* KO rats exhibited a significantly higher burst ratio than the WT rats (Figure 5A). Although the genotype × sex interaction was not significant [*F*(1, 31) = 1.60, *p* = 0.215], visual inspection of the data suggests the genotype main effect was driven primarily by the very low burst ratio in the WT females. The SHRs also had a higher burst ratio than the WKY rats (Figure 5B), although the main effect for strain did not quite exceed the criterion for significance [*F*(1, 44) = 3.89, *p* = 0.055]. Again, the effect of strain appears to be impacted by sex, (i.e., the SHR males had a higher burst ratio than the other groups), but the strain × sex interaction was not significant [*F*(1, 44) = 1.44, *p* = 0.237]. There were no significant genotype-related effects for peak location or peak area (Figure 5C, E). There were, however, strain-related effects on both of these parameters. For peak location, there was a main effect of strain [*F*(1, 44) = 305.35, *p* < 0.001], a main effect of sex [*F*(1, 44) = 76.337, *p* < 0.001], and a strain × sex interaction [*F*(1, 44) = 64.145, *p* < 0.001]. As seen in Figure 5D, the peak location of the SHR males and females occurred sooner than that of their same-sex WKY counterparts (*p* < 0.001 in both cases). In addition, the peak location for the SHR females occurred sooner than it did for the SHR males (*p* < 0.001). There was a similar trend in the SHR/WKY rats for peak area, but the strain × sex interaction was not significant [*F*(1, 44) = 2.48, *p* = 0.122]. Rather, there were only main effects for strain [*F*(1, 44) = 87.587, *p* < 0.001] and sex [*F*(1, 44) = 8.34, *p* = 0.006], which was lower in the SHRs and the females (Figure 5F).

### 3.2 | Delayed spatial alternation

#### 3.2.1 | Percent correct

**FIGURE 6:**
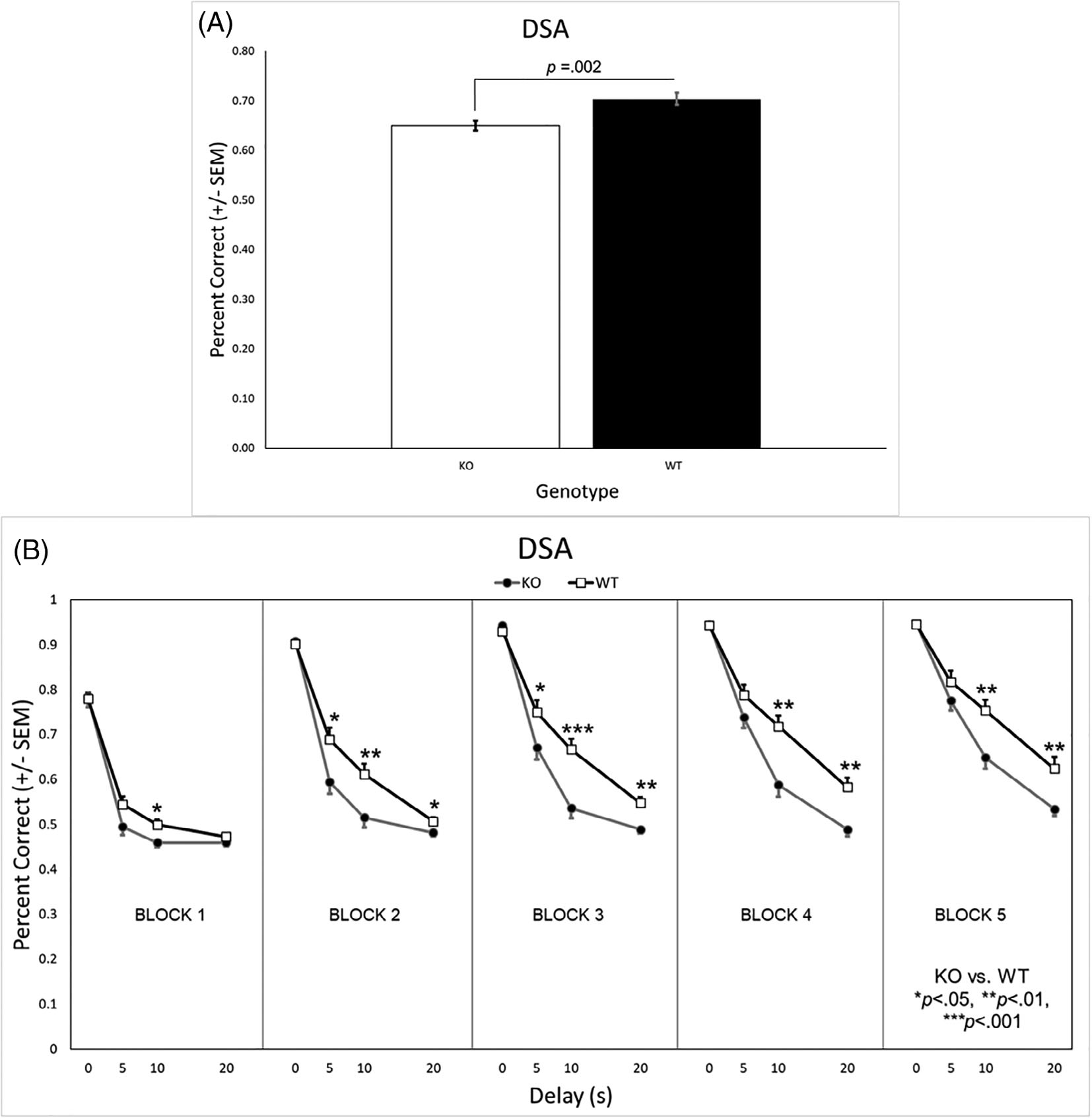
(A) WT rats had a significantly higher overall percentage correct on the DSA task that KO rats. (B) There were no differences on the DSA task when there was no delay. However, at the 5 s delay, WT rats had a higher percentage correct than KO rats during the second and third testing blocks (days 6–15) and when the delay was 10 s, a genotype difference was present during all testing blocks. While both genotypes were performing at chance during the first testing block (i.e., acquisition), when the delay was 20 s, the WT rats improved and were performing significantly better than the KO rats by the second testing block (days 6–10). DSA, delayed spatial alternation; KO, knockout; SEM, standard error of the mean; WT, wildtype

Analysis of the percent correct during DSA revealed a significant main effect of genotype [*F*(1, 31) = 11.48, *p* = 0.002], as well as a significant genotype × delay [*F*(1.7, 52.6) = 7.14, *p* = 0.003] and genotype × delay × block [*F*(6.9, 213.6) = 2.93, *p* = 0.006] interaction. The WT rats had a higher overall percent correct than the KO rats (Figure 6A). Post hoc analysis revealed no genotype differences at the 0 s delay (see Figure 5B). With a 5 s delay, however, WT rats had a higher percentage correct than KO rats during the second and third testing blocks. Increasing the delay to 10 s, resulted in a significant genotype difference during all testing blocks. Increasing the delay even more to 20 s, caused both groups to perform at chance levels during the first testing block where the KO rats remained for the remaining blocks. The WT rats, however, improved such that they were performing significantly better than the KO rats as early as the second testing block.

**FIGURE 7:**
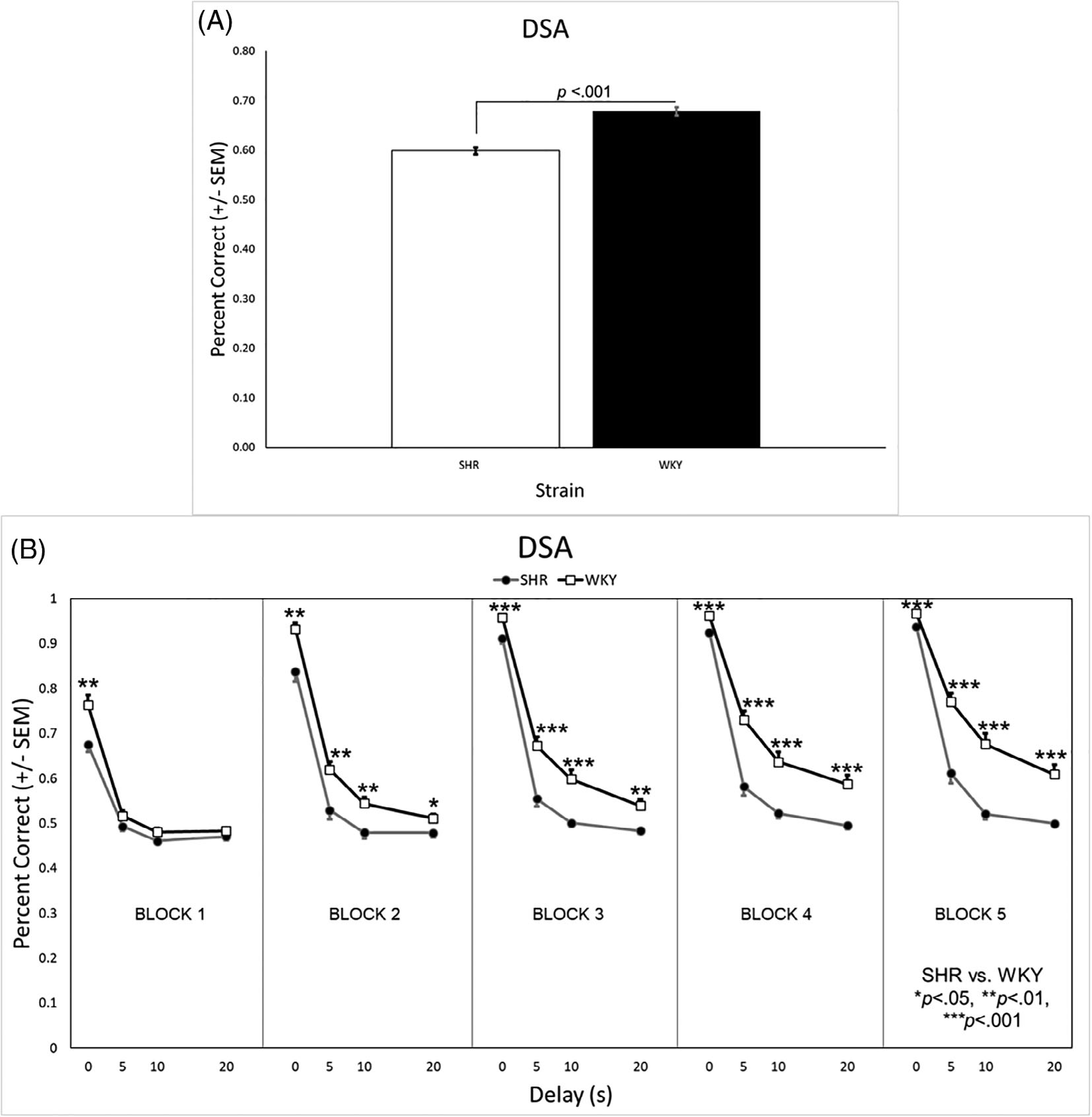
(A) WKY rats had a significantly higher overall percentage correct on the DSA task than SHRs. (B) The WKY rats had a higher percent correct across all blocks when the delay was 0 s. At the 5 s, 10 s, and 20 s delays, the WKY rats had a higher percent correct in all but the first testing block. DSA, delayed spatial alternation; SHR, spontaneously hypertensive rat; SEM, standard error of the mean; WKY, Wistar-Kyoto

Similar analysis in the SHR/WKY rats revealed a significant main effect of strain [*F*(1, 42) = 57.00, *p* < 0.001], as well as significant strain × block [*F*(2.4, 98.9) = 8.65, *p* < 0.001], strain × delay [*F*(2.2, 90.7) = 3.49, *p* = 0.031], and strain × delay × block [*F*(6.9, 292.2) = 9.74, *p* < 0.001] interactions. The WKY rats had a higher overall percent correct than the SHRs (Figure 6A). Post hoc analysis revealed the WKY rats outperformed the SHRs during all blocks when the delay was 0 s (see Figure 7B). When there was a 5, 10, or 20 s delay, the WKY rats had a higher percentage correct than SHRs in all but the first testing block.

#### 3.2.2 | Trial-by-trial analyses

**FIGURE 8:**
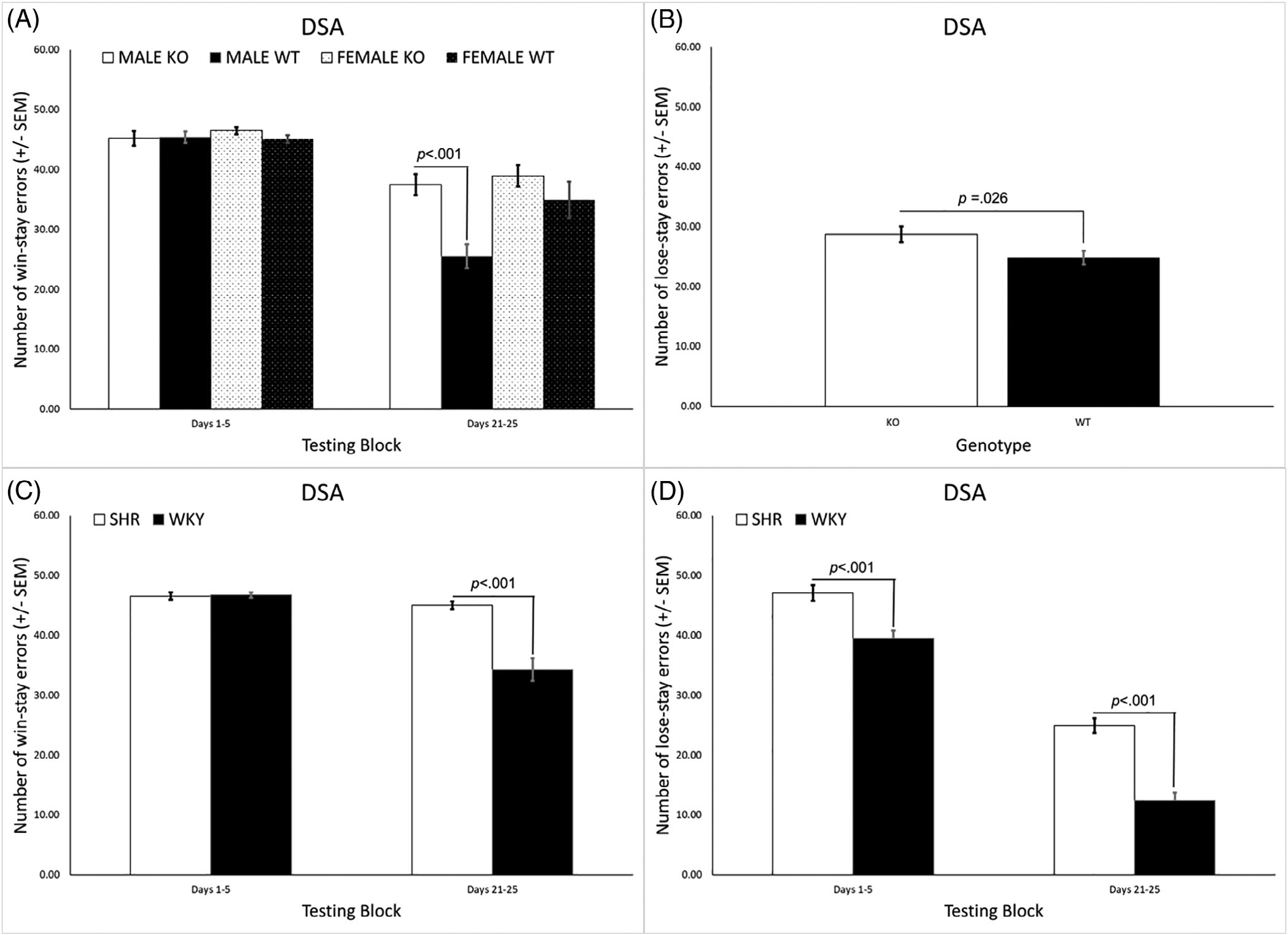
(A) KO rats committed a significantly larger overall number of win-stay errors compared with WT rats but this was primarily due to a difference between the male KO and WT rats during the last testing block (days 21–25). (B) KO rats also committed a significantly larger overall number of lose-stay errors compared with WT rats. (C) The SHRs had significantly more win-stay errors overall, primarily due to a difference from WKY rats during the last testing block (days 21–25). (D) The SHRs committed more lose-stay errors overall and during both the first and last testing block, with the difference from the WKY rats being larger during the latter (days 21–25). DSA, delayed spatial alternation; SHR, spontaneously hypertensive rat; SEM, standard error of the mean; WKY, Wistar-Kyoto

Analysis of win-stay errors in the first and last testing blocks revealed significant main effects of genotype [*F*(1, 31) = 11.125, *p* = 0.002] and sex [*F*(1, 31) = 5.271, *p* = 0.029] as well as significant genotype × block [*F*(1, 31) = 11.223, *p* = 0.002], sex × block [*F*(1, 31) = 5.091, *p* = 0.031], and genotype × sex × block [*F*(1, 31) = 4.682, *p* = 0.038] interactions. KO rats had significantly more win-stay errors overall, but this was primarily due to a difference between the male KO and WT rats during the last testing block (days 21–25; Figure 8A). Analysis of lose-stay errors revealed a significant main effect of genotype [*F*(1, 31) = 5.497, *p* = 0.026] and a significant sex × block interaction [*F*(1, 31) = 7.591, *p* = 0.010]. KO rats committed more lose-stay errors overall (Figure 8B). In addition, while the number of lose-stay errors was similar for males and females during block 1 (40.9 ± 1.9 and 40.1 ± 1.3, respectively), males made significantly fewer lose-stay errors than females during block 5 (9.9 ± 1.4 and 15.8 ± 1.5, respectively).

In the SHR/WKY rats the analysis of win-stay errors revealed significant main effects of strain [*F*(1, 42) = 27.064, *p* < 0.001] and sex [*F*(1, 42) = 4.750, *p* = 0.035] and a significant strain × block interaction [*F*(1, 42) = 26.484, *p* < 0.001]. SHRs had significantly more win-stay errors overall, primarily due to a difference from WKY rats during the last testing block (days 21–25; Figure 8C). Analysis of lose-stay errors revealed a main effect of strain [*F*(1, 42) = 61.398, *p* < 0.001] and a significant strain × block interaction [*F*(1, 42) = 4.4085, *p* = 0.042]. SHRs committed more lose-stay errors overall and during both the first and last testing block, with the difference from the WKY rats being larger during the last testing block (Figure 8D).

## 4 | DISCUSSION

Evidence of behavioral disinhibition was present in both the *Lphn3* KO rats as well as the SHRs. Relative to their controls, *Lphn3* KO rats and SHRs exhibited impaired inhibitory control performance during the DRL tasks due to a higher proportion of responses with short IRTs that was accompanied by a lower proportion of responses with longer IRTs, the latter of which were necessary to be earn a reinforcer. Likewise, the burst ratio was higher in the *Lphn3* KO rats and SHRs, although these effects appeared to be differentially modulated by sex. In the case of genotype, the effect was driven by the lower burst ratio in the female WT rats, while for strain, the SHR males had a higher burst ratio than the other groups. Looking across DRL 15 testing blocks, the SHRs appear to be more profoundly affected than the *Lphn3* KO rats (Figure 1A, C). This interpretation is based not only on the smaller KO vs. WT difference (Figure 1B) in comparison with the SHR vs. WKY difference (Figure 1D), but also by directly comparing the ratio of reinforced: nonreinforced responses of the KO rats to the SHRs (white bars in Figures 1B, D). Furthermore, the *Lphn3* KO rats were able to earn an equal number of reinforcers as WT rats during DRL 15 by compensating for their decreased efficiency by completing a larger number of trials. This was not the case for the SHRs, who also had decreased efficiency, but were not able to earn an equivalent number of reinforcers even when completing more trials. In other words, while the *Lphn3* KO rats were able to work harder to overcome their efficiency deficit, the SHR rats – especially the females - were not. Lastly, no genotype effects were seen for peak location or peak area, while the peak location was earlier and peak area was lower in the SHRs, especially the SHR females.

Working memory deficits were also present in both the *Lphn3* KO rats and SHRs, with the relative degree of impairment again being greater in the SHRs. During NCA (Supplemental Figure 9) and the 0 s delay of DSA, the *Lphn3* KO rats did not differ from the WT rats. Rather, persistent differences between the genotypes were evident only during the longer 10 and 20 s DSA delays (Figure 6). This was not the case for the SHRs, who had significantly poorer performance than the WKY rats across all delays (0, 5, 10, and 20 s) of DSA (Figure 7) and even during NCA (Supplemental Figure 9). This discrepancy is also apparent by directly comparing overall DSA performance of the *Lphn3* KO rats with the SHRs (white bars in Figures 6A, 7A).

## 5 | RULING OUT A MOTOR IMPAIRMENT

Importantly, the DRL and DSA impairments exhibited by the *Lphn3* KO rats and SHRs do not represent a motor impairment. During DRH, when the response requirement was high, the *Lphn3* KO rats and SHRs were not impaired. In fact, the *Lphn3* female KO rats earned more reinforcers than the WT females. Similarly, the SHRs (no sex difference) earned more reinforcers and exhibited greater DRH efficiency than the WKY rats (Supplemental Figure 1). Likewise, during CA (Supplemental Figure 8) the *Lphn3* KO rats and SHRs were able to alternate levers by following the cue light just as effectively as their WT and WKY counterparts, respectively.

### 5.1 | Response acquisition versus steady-state responding

During DRL, the difference in the ratio of reinforced: nonreinforced trials between the *Lphn3* KO and WT rats was relatively consistent across testing blocks (Figure 1A) such that the difference between the strains was manifested only as a main effect of genotype (Figure 1B). In other words, the genotypes learned at a similar *rate* across blocks, but the WT rats performed consistently better. The ratio of reinforced: nonreinforced trials for the WKY rats rose sharply across testing blocks compared with the SHRs who showed only a modest increase in performance across testing blocks. Steady-state performance of the WKY rats was much higher than the SHRs as well. Said differently, the WKY rats learned to complete the DRL 15 task faster and better than the SHRs (Figure 1C) yielding higher performance overall (Figure 1D).

The above pattern was also prevalent during DSA. During the first block, both the *Lphn3* KO and WT rats were at (or slightly above) chance performance when any delay (5, 10, or 20 s) occurred. Both genotypes improved across testing blocks when presented with the 5, 10, and 20 s delays, with the relative difference observed between the WT and KO rats being fairly consistent across blocks (Figure 6B). Again, there was no obvious difference in the rate of learning between the genotypes, but there was a large discrepancy in *how well* the two genotypes learned the task. On the other hand, the WKY rats increased their performance across testing blocks to a greater degree than the SHRs, whose performance did not change much across testing blocks, particularly at the longer delays (Figure 7B). As was the case during DRL, SHRs exhibited both acquisition and steady-state performance deficits on DSA compared with their WKY control strain.

### 5.2 | Utility of the *Lphn3* KO rat as an animal model of ADHD

Like the SHRs, the *Lphn3* KO rats exhibited deficits in inhibitory control and working memory, albeit the relative degree of impairment was greater in the SHRs. Inhibitory control and working memory are part of a collection of top-down, higher-order cognitive processes known as executive functions (EF).^66^ Attention is also an EF,^66^ that is often impaired in individuals with ADHD.^67^ Notably, ADHD is characterized by two primary presentations - hyperactivity/impulsivity and inattention – which can occur individually or together, thereby resulting in three unique ADHD sub-types: hyperactive/impulsive, inattentive, or combined.^1^ The SHRs appear to represent the combined ADHD sub-type as they are hyperactive, impulsive, and inattentive. ^57,68^ On the other hand, data collected to date, including the current study and others that have used *Lphn3* KO models,^35–38,69,70^ only provide evidence for the hyperactive/impulsive subtype. Direct measures of attention are limited, but suggest it is not affected following *Lphn3* deletion. For example, Mortimer et al.^35^ used a touchscreen operant response to assess *Lphn3* KO and WT mice on a continuous performance task. If mice pressed the screen when the target was presented, they received a food reinforcer and the response was classified as a “hit”. If they failed to press when the target was presented (i.e., they omitted a trial), this response was classified as a “miss.” If the *Lphn3* KO mice had an attentional deficit, this would have increased the number of misses. This was not the case, however, as Mortimer and colleagues reported that the hit rate (i.e., hits / [hits + misses]) did not differ between the genotypes.

The current study provides indirect evidence on attention. While not a direct measure of attention, the NCA task has a minimal working memory requirement and can provide cursory information about the ability to “stay on task.” While there were no effects of genotype on NCA performance, this was not the case in the SHRs who exhibited a performance decrement across all 10 days of NCA (see Supplemental Figure 8). Ongoing research is assessing attention in SHRs and *Lphn3* KO rats more directly and comparing their data with WKY and *Lphn3* WT rats to better assess whether the two former exemplify different ADHD subtypes.

### 5.3 | *Lphn3*, executive function, and catecholamines

Proper modulation of catecholamine neurotransmission is essential for guiding optimal prefrontal cortex (PFC) function – the neural structure that gives rise to EF.^71^ *Lphn3* gene expression affects catecholamine neurotransmission. Research on zebrafish larvae has demonstrated down-regulation of *lphn3.1* (one of the two zebrafish *lphn3* orthologs) resulted in a decrease and misplacement of dopamine (DA) (but not norepinephrine or serotonin) neurons,^38^ as well as decreased locomotor sensitivity to DA agonists and antagonists.^70^ The hyperactivity seen in *lphn3.1* mutant larvae was rescued by the commonly prescribed ADHD catecholamine-regulating medications: methylphenidate and atomoxetine.^38^ Compared with WT mice, *Lphn3* mutant mice had increased differential gene expression of DA receptor D4 (DRD4), serotonin receptor 2a (5-HT2A), the serotonin transporter (5-HTT), and tyrosine hydroxylase (TH; required for the synthesis of DA) on P0, as well as increased striatal DA and serotonin when 4–6 weeks of age.^36^ In *Lphn3* KO rats,^37^ no changes in the levels of DA, norepinephrine, serotonin, or their major metabolites were seen in striatum, hippocampus, or PFC when measured by HPLC. Likewise, the expression of DRD2, DRD4, and the vesicular monoamine transporter (VMAT-2) did not differ between the genotypes. However, TH and aromatic L-amino acid decarboxylase (AADC) were increased, while DRD1 was decreased,^37^ suggesting greater DA and/or NE availability.

In *Lphn3* mutant mice and rats, there are differential expression levels of the DA transporter (DAT) gene (*Slc6a3*) and the protein itself. Adult *Lphn3* KO rats have increased DAT expression^37^ and increased reuptake of DA (i.e., functional implication for increase in DAT)^72^ in the dorsal striatum. Likewise, adult *Lphn3*^−*/*−^ mice demonstrate overexpression of *Slc6a3* in whole brain^36^ and in the dorsal striatum,^34^ but downregulation of *Slc6a3* in the PFC.^35,69^ DAT expression is associated with ADHD, cocaine addiction, and alcoholism (see ref. [73] for review] making these results noteworthy.

The exact mechanism whereby alterations in *Lphn3* gene expression alters catecholamine neurotransmission is still being investigated. LPHN3 is an adhesion G-protein coupled receptor (GPCR) that is expressed in the brain.^74,75^ It is more robustly expressed early in development, while expression decreases as the brain matures.^23^ The LPHN3 receptor binds endogenous ligand fibronectin and leucine-rich repeat transmembrane protein 3 (FLRT3).^76^ FLRT3 is involved in cell adhesion, neuronal signaling, and synaptic organization, and disruptions in FLRT3 function lead to reductions in excitatory synapse density and a weakened afferent input.^69^ The interaction between LPHN3 and FLRT3 plays an important role in transmembrane signaling, Ca^2+^ mobilization from presynaptic stores, neuronal structure, and synaptic development.^69,77,78^ The LPHN3 receptor also has the ability to interact with SHANK proteins,^79^ which is significant as interference in SHANK protein functioning is linked to abnormal structural changes in dendritic spines and excitatory synapses, as well as defects in corticostriatal circuitry.^80–82^

### 5.4 | A Role for *Lphn3* in EDs

As previously mentioned, impulsivity is a behavior that is common to EDs including ADHD and SUD^1,7,39–41^ and deficits in working memory are believed to contribute to impulsivity.^42,43^ The current results demonstrate *Lphn3* expression is involved in regulating response inhibition and working memory. Numerous studies have implicated *Lphn3* in the etiology of ADHD.^17,20–24,26–32^ While less is known about whether *Lphn3* contributes to the cause of other EDs, there is some evidence suggesting this is the case.^13,25,33,69^ Future research in our lab will assess whether differences in drug self-administration (as a proxy measure for SUD) exist between *Lphn3* KO and WT rats. Given the high degree of comorbidity^1,5–7^ and shared heritability^12,15^ of EDs, as well the role that *Lphn3* plays in neurodevelopment, it seems plausible that *Lphn3* may be part of a shared mechanism.

## Supplementary Material

Supplementary materials

## Data Availability

The data that support the findings of this study are available from the corresponding author upon reasonable request.
