# Supplementary material for: An assessment of executive function in two different rat models of attention-deficit hyperactivity disorder: Spontaneously hypertensive versus Lphn3 knockout rats
Source: Genes Brain Behav. Author manuscript; Available in PMC 2023 Apr 19. (PMC10114166; doi:10.1111/gbb.12767)
Supplement: Supplementary materials [file NIHMS1887119-supplement-Supplementary_materials.pdf]

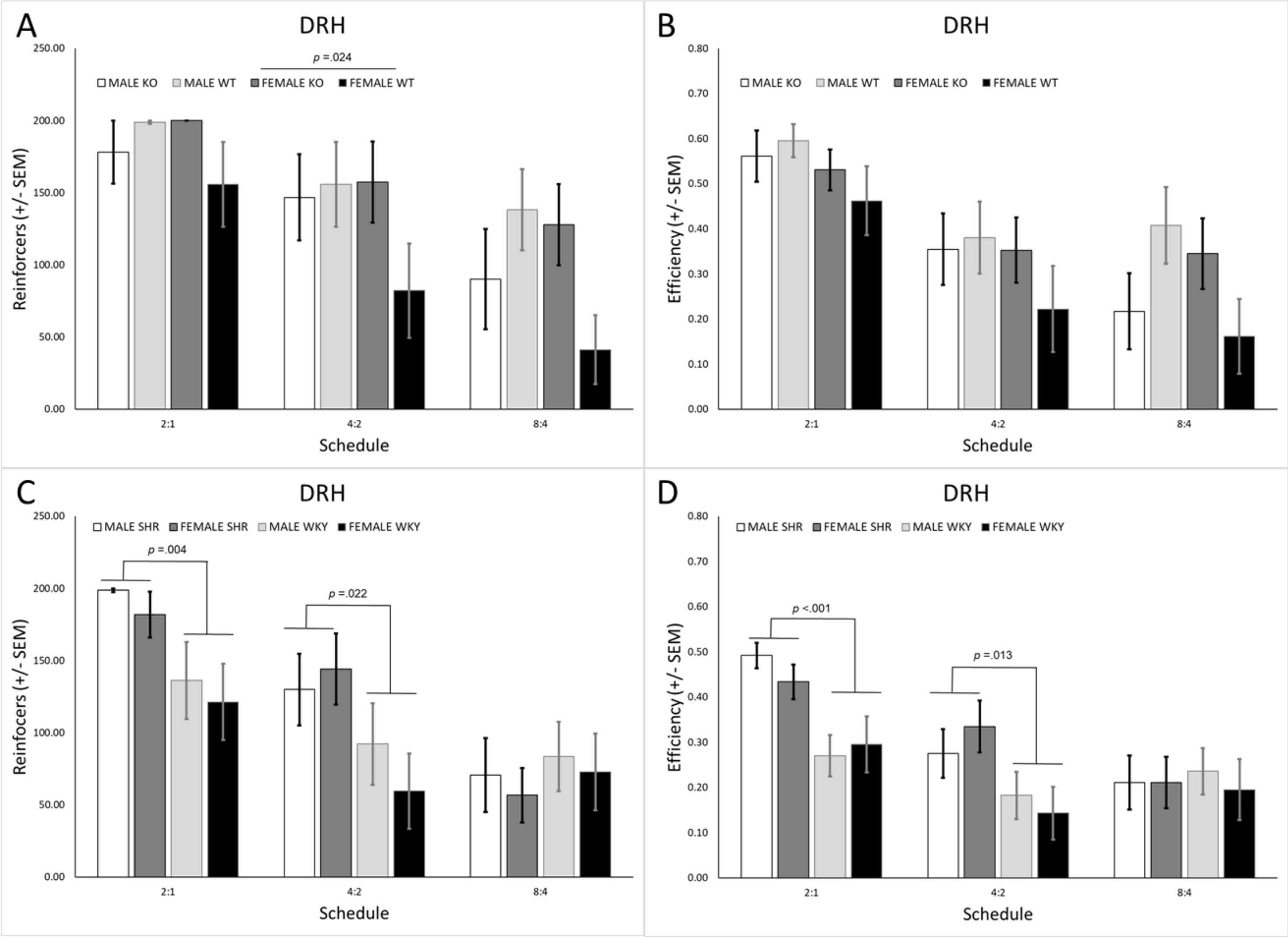

Supplemental Figure 1. No significant genotype- or strain-related findings were found for number of lever presses during DRH. **(A)** There was a significant genotype x sex interaction for reinforcers earned, $F(1,31) = 5.711$, $p = .024$. Additional post hoc analysis averaged across all DRH schedules indicated female KO rats earned significantly more reinforcers than WT females ($p = .024$) with no genotype difference in the males. **(B)** There was also a significant genotype × sex interaction for efficiency [$F(1,31) = 5.045$, $p = .032$] but post hoc analysis averaged across all DRH schedules found no significant effect of genotype for either the males or females. **(C)** There was a significant strain × schedule interaction on the number of reinforcers earned, $F(1.962,86.335) = 6.929$, $p = .002$. SHRs earned significantly more reinforcers than WKY rats during DRH 2:1 ($p = .004$) and 4:2 ($p = .022$). **(D)** There was also significant main effect of strain [$F(1,44) = 5.897$, $p = .019$] and strain × schedule interaction for DRH efficiency, $F(1.882,80.178) = 6.541$, $p = .003$. SHRs were more efficient than WKY rats during DRH 2:1 ($p = .004$) and 4:2 ($p = .022$). DRH=Differential Reinforcement of High Rates, KO = knockout, WT=wildtype, SHR=spontaneously hypertensive rat, WKY=Wistar-Kyoto, SEM=standard error of the mean, 2:1 = 2 lever presses in 1 s, 4:2 = 4 lever presses in 2 s, 8:4 = 8 lever presses in 4 s

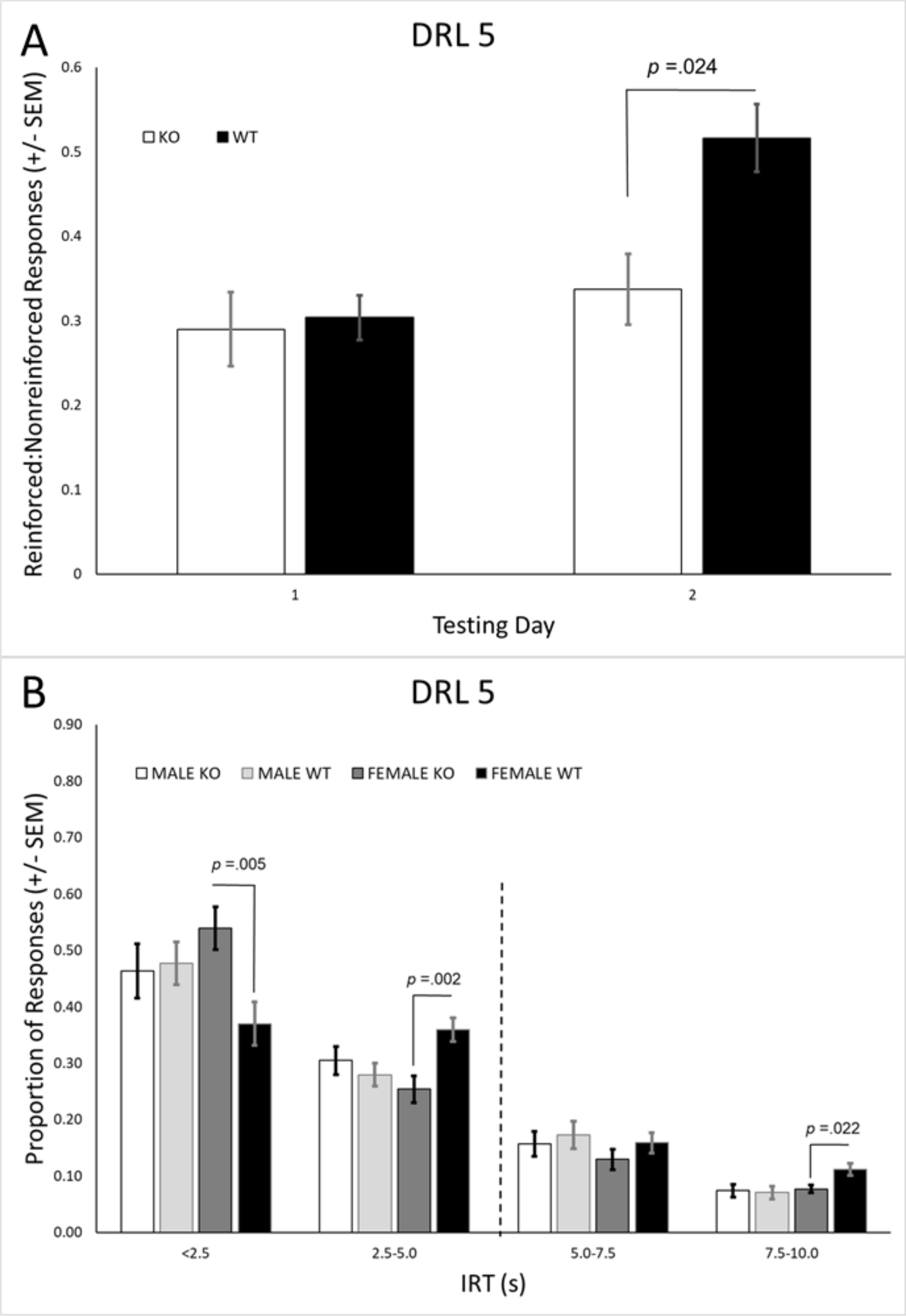

Supplemental Figure 2. (A) There was a significant genotype × day interaction on the ratio of reinforced to nonreinforced responses for DRL 5, $F(1,31) = 4.625$, $p = .039$. Additional post hoc analysis revealed a difference between *Lphn3* KO and WT rats only on day 2 ($p = .024$). **(B)** There was a significant genotype × sex × IRT interaction, $F(1.448,44.886) = 4.635$, $p = .024$. Simple effects analysis within each IRT bin revealed a significant effect of group in the <2.5 s ($p = .041$), 2.5-5.0 s ($p = .015$), and 7.5-10.0 s ($p = .026$) IRT bins. In all three of these IRT bins, there was a significant difference between the female genotypes. Female KO rats exhibited a higher proportion of responses in the <2.5 s IRT bin, but a lower proportion of responses in the 2.5-5.0 and 7.5-10.0 s bins compared with the WT females. DRL=Differential Reinforcement of Low Rates, KO = knockout, WT=wildtype, IRT = inter-response time, SEM=standard error of the mean

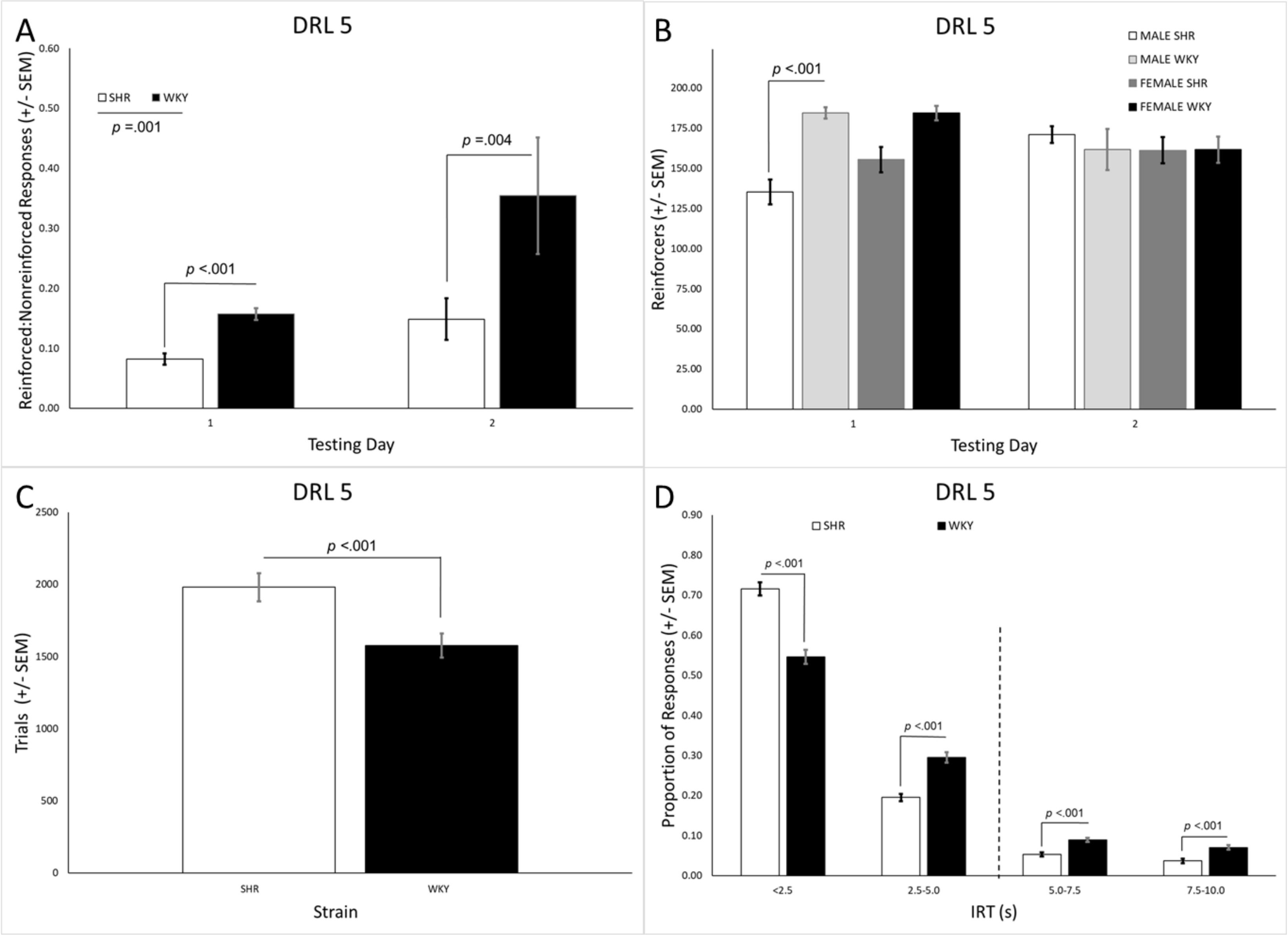

Supplemental Figure 3. (A) There were significant main effect of strain [$F(1,44) = 13.370$, $p = .001$] as well as a significant strain × day interaction [$F(1,44) = 4.474$, $p = .040$] on the ratio of reinforced to nonreinforced responses for DRL 5. WKY rats had a higher ratio on both days. **(B)** Analysis of reinforcers earned revealed a main effect of strain [$F(1,44) = 7.253$, $p = .010$] and significant interactions of strain × day [$F(1,44) = 11.276$, $p = .002$] and strain × sex × day [$F(1,44) = 11.308$, $p = .002$]. Simple effects analysis for each day revealed a significant effect of group only on day 1 ($p<.001$), wherein the WKY males but not WKY females earned more reinforcers than the same-sex SHRs. **(C)** There was a significant main effect of strain on the number of trials completed, $F(1,44) = 46.704$, $p = .001$. **(D)** There was a significant strain × IRT interaction, $F(1.440,63.372) = 35.55$, $p < .001$. SHRs exhibited a significantly higher proportion of responses in the shortest IRT bin (<2.5 s), but a lower proportion in all other IRT bins. DRL=Differential Reinforcement of Low Rates, IRT = inter-response time, SHR=spontaneously hypertensive rat, WKY=Wistar-Kyoto, SEM=standard error of the mean

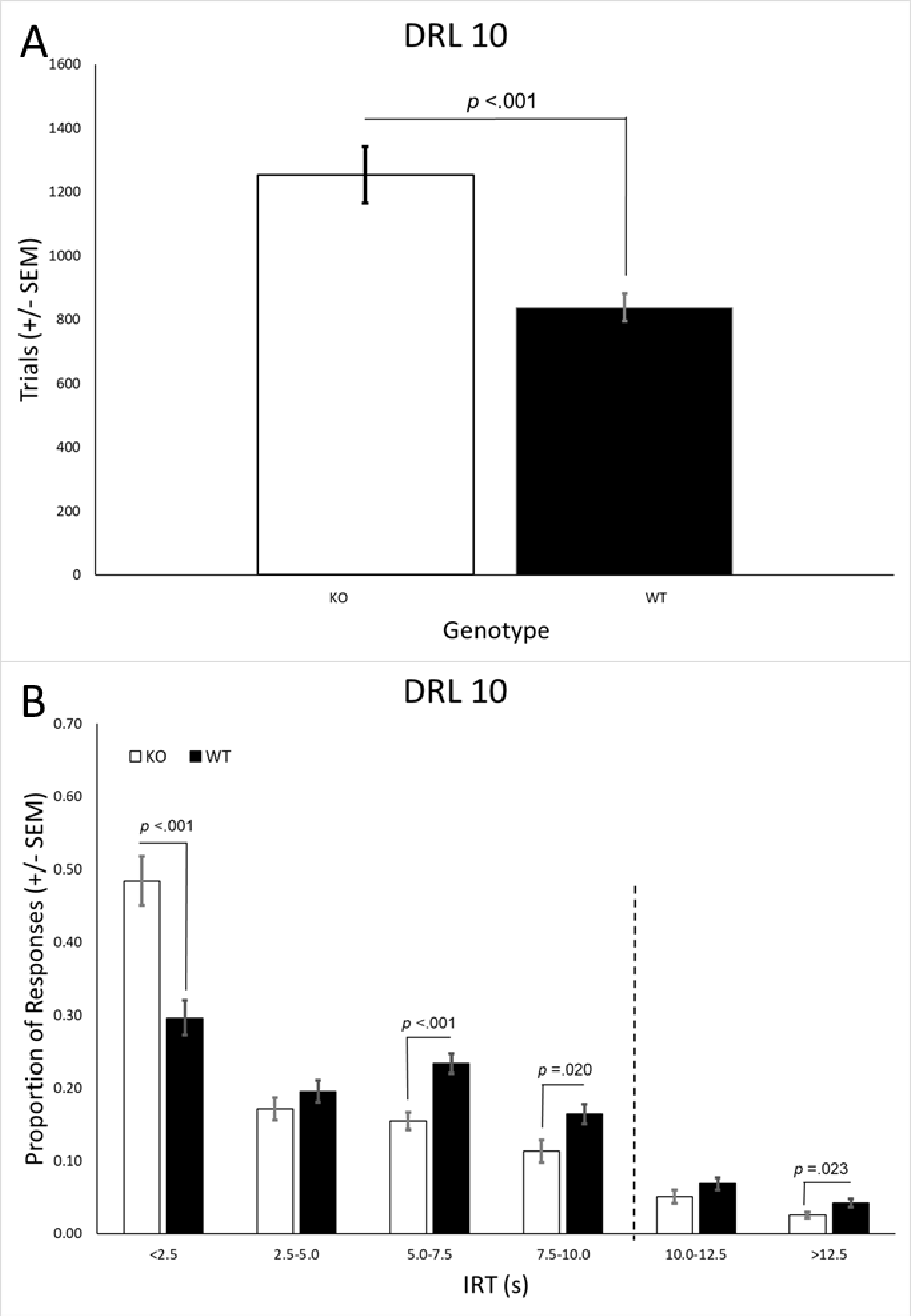

Supplemental Figure 4. (A) The main effect for genotype was significant for the number of trials completed during DRL 10, $F(1,31) = 18.783$, $p < .001$. The *Lphn3* KO rats completed significantly more trials than the WT rats. **(B)** There was a significant genotype × IRT interaction, $F(1.843,57.145) = 13.971$, $p < .001$. Post hoc analysis within each IRT bin revealed a significant difference between the *Lphn3* KO and WT rats in the <2.5 s, 5.0-7.5 s, 7.5-10.0 s, and 12.5-15.0 s IRT bins. The KO rats exhibited a significantly higher proportion of burst responding during the shortest (i.e., <2.5 s) IRT bin while the proportion of responses by the KO rats was significantly lower than the WT rats in the other IRT bins that exhibited a genotype difference. DRL=Differential Reinforcement of Low Rates, KO = knockout, WT=wildtype, IRT = inter-response time, SEM=standard error of the mean

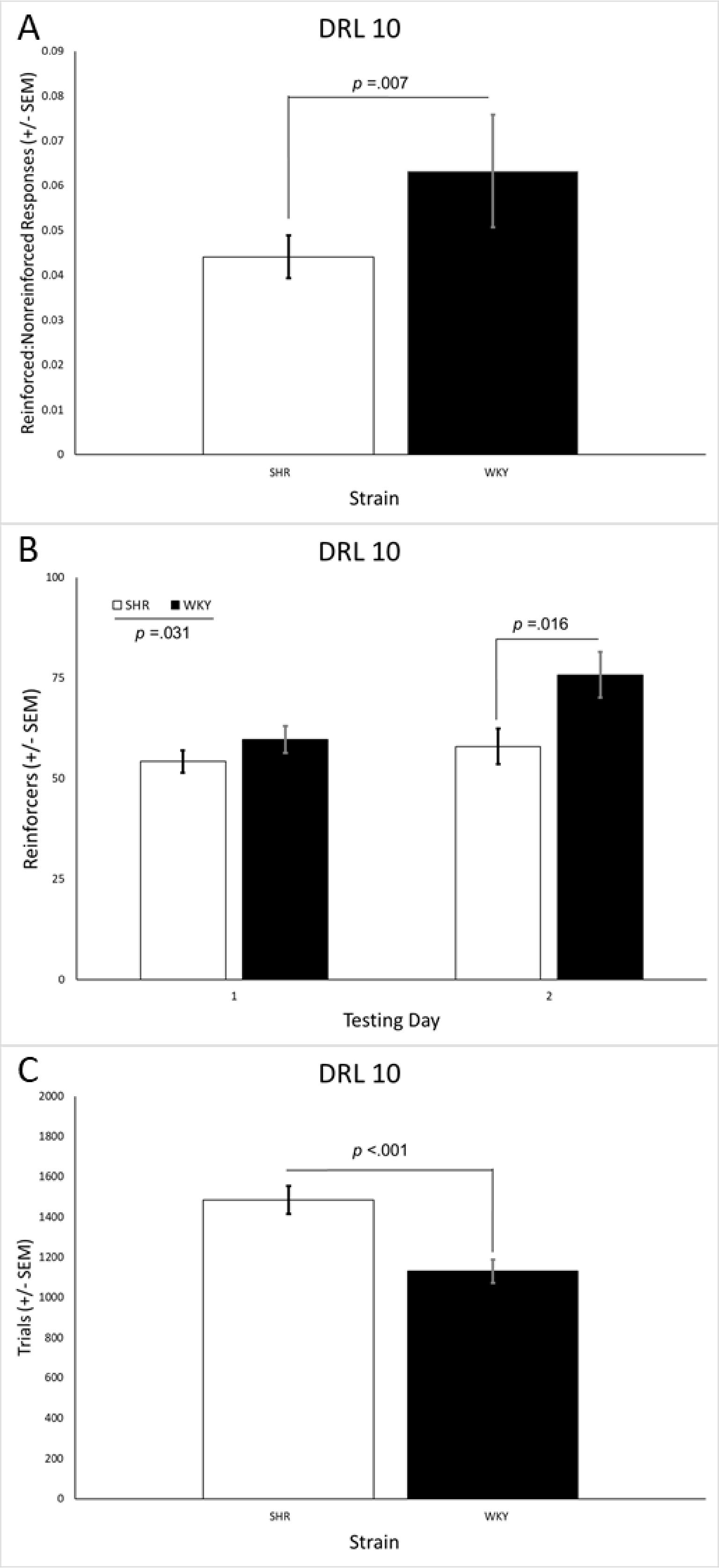

Supplemental Figure 5. (A) The main effect for strain was significant for the ratio of reinforced to nonreinforced trials during DRL 10, $F(1,44) = 8.009$, $p = .007$. The WKY rats had a higher ratio than the SHRs. (**B)** There was a significant main effect of strain [$F(1,44) = 4.964$, $p = .031$] and significant strain × day interaction [$F(1,44) = 4.648$, $p = .037$]. Post hoc analysis revealed the WKY rats earned more reinforcers than the SHRs on day 2. **(C)** The main effect for strain was significant for number of trials completed, $F(1,44) = 15.027$, $p < .001$. The WKY rats completed fewer trials than the SHRs. DRL=Differential Reinforcement of Low Rates, SHR=spontaneously hypertensive rat, WKY=Wistar-Kyoto, SEM=standard error of the mean

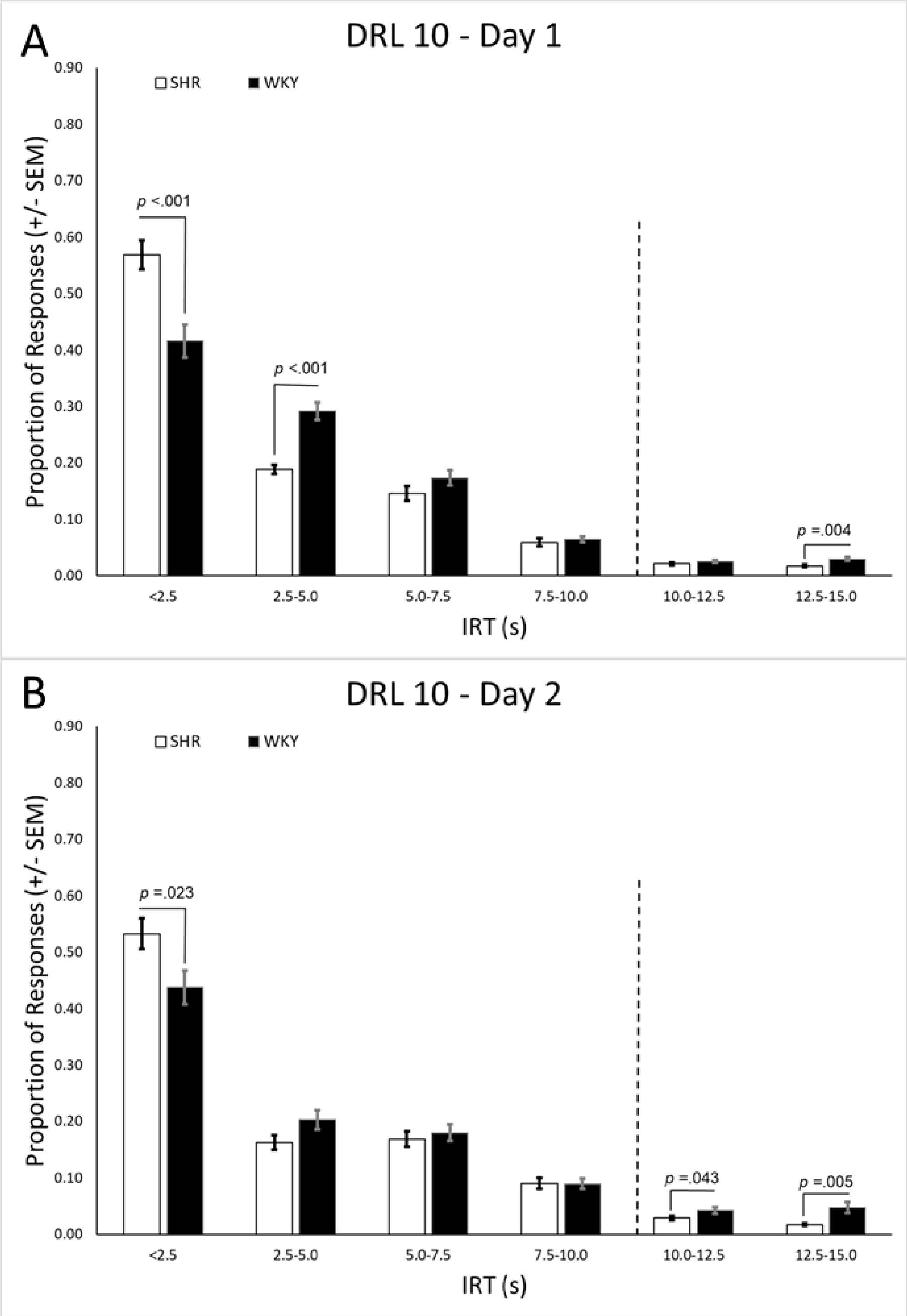

Supplemental Figure 6. The strain × IRT [$F(1.342,59.033) = 9.257$, $p = .001$] and strain × day × IRT [$F(2.306,101.449) = 80.169$, $p < .001$] interactions were significant . **(A)** Post hoc analysis within each IRT bin on each day revealed a significant difference between the stains in the <2.5 s, 2.5-5.0 s, and 12.5-15.0 s IRT bins on day 1. **(B)** On day 2, post hoc analysis revealed a significant difference between the strains in the <2.5 s, 10.0-12.5 s, and 12.5-15.0 s IRT bins. The SHR rats exhibited a greater proportion of burst responding during the first IRT bin on both days as well as a lower proportion of responses during the later bins that were reinforced. DRL=Differential Reinforcement of Low Rates, SHR=spontaneously hypertensive rat, WKY=Wistar-Kyoto, IRT = inter-response time, SEM=standard error of the mean

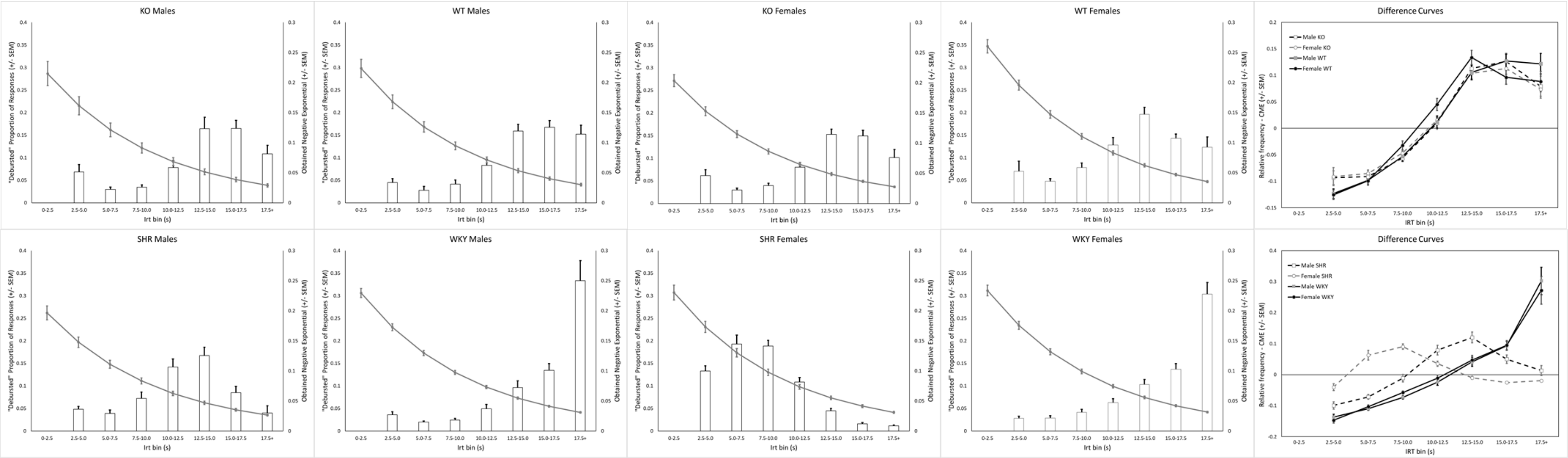

Supplemental Figure 7. The "debursted" relative proportion of responses for the pause IRTs (i.e., IRTs > 2.5 s) with the negative exponential curves, as well as graphs showing the difference curves for the different genotypes (top row) and different strains (bottom row). The figures on the right side of each row were used to determine the peak location and peak area for the last testing block of DRL 15. DRL=Differential Reinforcement of Low Rates, KO = knockout, WT=wildtype, SHR=spontaneously hypertensive rat, WKY=Wistar-Kyoto, IRT = inter-response time, SEM=standard error of the mean

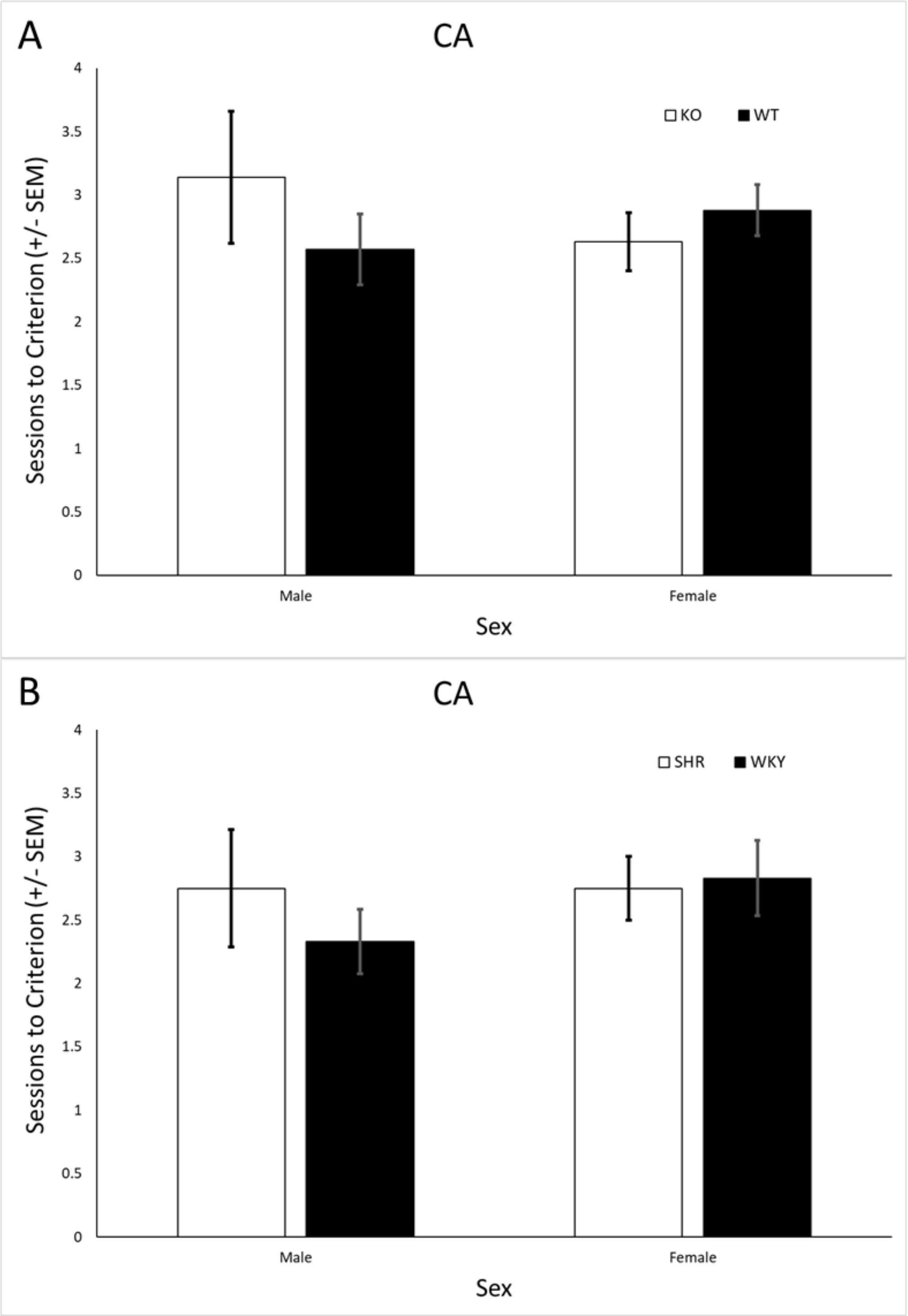

Supplemental Figure 8. The number of sessions required to reach criterion performance for CA did not differ based on **(A)** genotype (KO vs. WT) or **(B)** strain (SHR vs. WKY). There were no sex-related differences either. KO = knockout, WT=wildtype, SHR=spontaneously hypertensive rat, WKY=Wistar-Kyoto, SEM=standard error of the mean

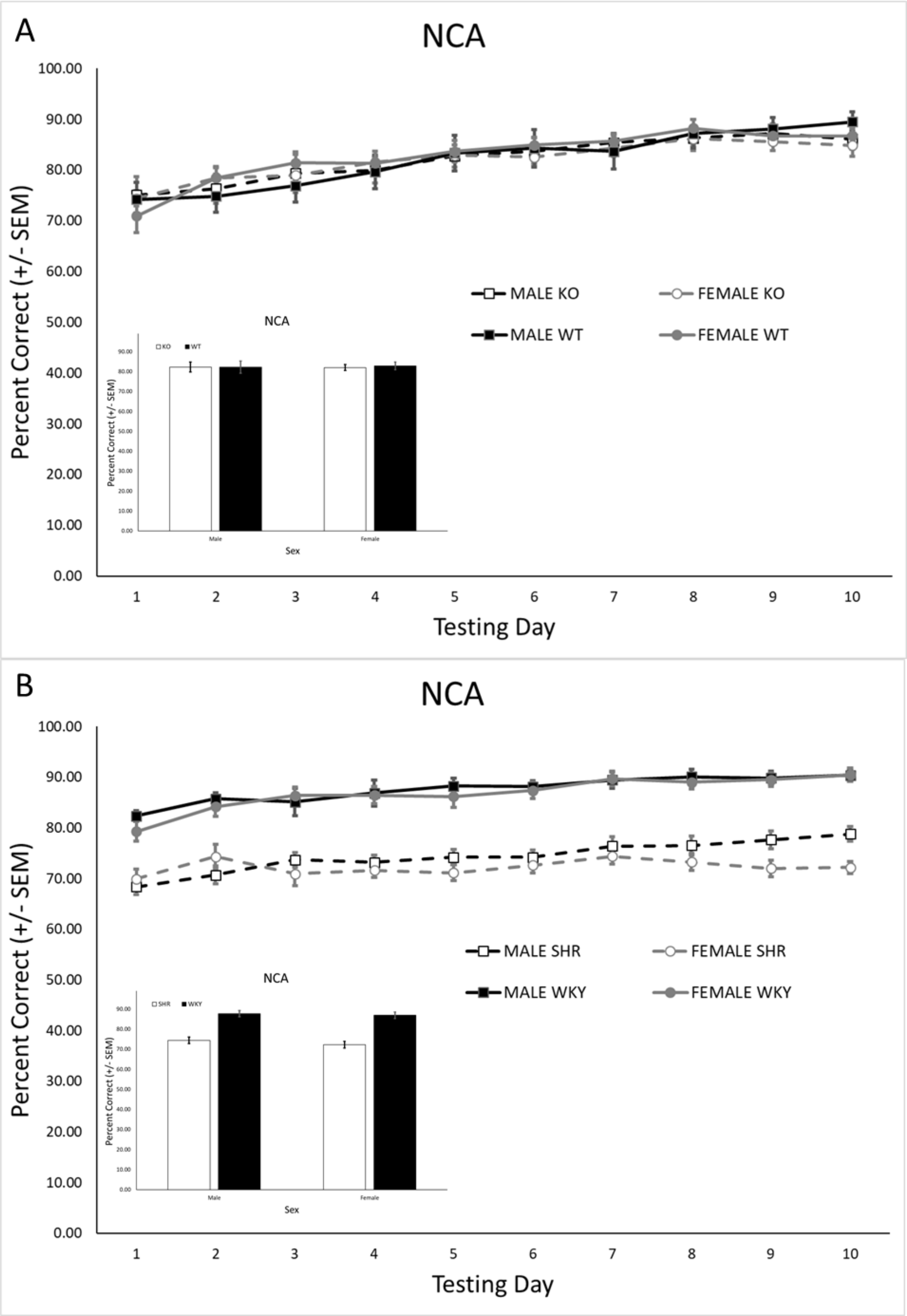

Supplemental Figure 9. There were no differences in the percent correct on the NCA task based on **(A)** genotype (*Lphn3* KO vs. WT; inset) or genotype on any testing day. However, there was a significant difference in the percent correct on the NCA task based on **(B)** strain ($F(1,42) = 151.945$, $p < .001$) whereby the WKY rats exhibited better performance than the SHRs across all 10 days of NCA. There were no sex-related effects. KO = knockout, WT=wildtype, SHR=spontaneously hypertensive rat, WKY=Wistar-Kyoto, NCA = non-cued alternation, SEM=standard error of the mean
